# Depth-Dependent Characterization of Vertical Cracks in Concrete Using Lamb Wave Active Sensing

**DOI:** 10.3390/s26144563

**Published:** 2026-07-18

**Authors:** Nontawat Srisapan, Theophilus Asumah, Roohollah Askari

**Affiliations:** Department of Geological and Mining Engineering and Sciences, Michigan Technological University, 1400 Townsend Dr, Houghton, MI 49931, USA; taasumah@mtu.edu (T.A.); raskari@mtu.edu (R.A.)

**Keywords:** Lamb waves, controllable mechanical source, vertical crack, crack characterization, phase-velocity anisotropy, quality factor, NDT, SHM

## Abstract

**Highlights:**

**What are the main findings?**

- A repeatable tunable high-frequency mechanical source is used for concrete crack sensing.
- Phase velocity and quality factor show directional dependence across a concrete crack.

**What are the implications of the main findings?**

- Frequency bandwidth is tuned by varying tip stiffness, tip weight, duty cycle, and impact force.
- Phase-velocity anisotropy ratio depends on crack depth.

**Abstract:**

Vertical cracks in concrete present a major challenge for many conventional nondestructive testing methods (NDT) and structural health monitoring (SHM) methods. Elastic wave-based approaches offer strong interaction with crack faces and depth sensitivity; however, their effectiveness is often limited by the lack of repeatable and tunable excitation sources. Repeatability is critical because scattered and attenuated signals require stacking to achieve adequate signal-to-noise ratios, while tunability is essential because key crack attributes are frequency-dependent and must be probed at appropriate wavelengths. In this study, we develop an active sensing system utilizing a linear impact actuator as a repeatable and tunable mechanical source for elastic-based NDT and apply it to a 0.24 m thick concrete slab containing three surface-breaking vertical cracks with depths of 6, 12, and 18 cm, respectively. The actuator is tuned by adjusting impact conditions to generate $A_{0}$-dominated Lamb wave responses. For each crack, two linear arrays are deployed, one parallel and one perpendicular to the crack trace, to investigate directional anisotropy. Phase-velocity anisotropy is quantified using the $A_{0}$ Lamb wave dispersion curves, while the effective quality factor is used as a complementary indicator of direction-dependent attenuation. Our results show that phase velocities are consistently higher for crack-parallel propagation than for crack-perpendicular propagation, and that the degree of anisotropy increases with crack depth. The quality factor decreases with increasing crack depth and exhibits anisotropic behavior, with systematically lower values for crack-perpendicular measurements compared to crack-parallel measurements. Overall, the results demonstrate that controllable and repeatable impact excitation establishes a reliable framework for elastic-wave-based characterization of idealized vertical cracks in concrete.

## 1. Introduction

Vertical cracks in concrete are a critical form of damage in civil infrastructure because they can compromise structural integrity, facilitate moisture ingress, accelerate reinforcement corrosion, and evolve into more severe failures if left undetected [1,2,3]. Despite their importance, vertical cracks remain challenging to characterize using many conventional nondestructive testing (NDT) and SHM. Various active and passive methods have been developed to identify and characterize vertical cracks. Infrared thermography is a passive sensing method where surface temperature variations caused by differences in heat transfer are measured to detect subsurface cracks and material anomalies. However, the method is primarily sensitive to near-surface cracks and provides limited information on subsurface crack depth and geometry [4,5,6]. Ground-Penetrating Radar (GPR) is an active sensing approach where cracks are detected through contrasts in electromagnetic properties that generate reflections and scattering of radar waves. GPR may exhibit reduced sensitivity to vertical cracks due to unfavorable orientation and weak contrast [7,8,9,10]. Another passive approach is the acoustic emission technique, where transient elastic waves generated by crack initiation and propagation are detected and analyzed to identify material damage. Such method depends on active damage processes and do not directly image existing cracks [11]. In contrast, elastic-wave-based approaches offer strong interaction with crack faces and depth sensitivity, making them well-suited for probing subsurface cracks [12,13]. Recent hybrid methods combining active guided-wave techniques with passive acoustic emission monitoring have also demonstrated comprehensive SHM assessments of deterioration, particularly in complex reinforced elements [14]. In addition to NDT techniques, modern field assessments increasingly deploy artificial intelligence and computer vision to evaluate surface morphology. For instance, Wu et al. [15] introduced deep learning frameworks utilizing lightweight semantic segmentation networks combined with depth camera imaging to achieve precise pixel-level measurements of crack widths in dams. Similarly, recent advancements have leveraged robotic platforms fusing high-precision LiDAR and depth cameras for sub-millimeter 3D mapping of external surface cracks [16]. While these optical and machine learning tools represent the state of the art for automating surface aperture quantification and external contour recognition, capturing the subsurface profile and penetration depth of structural damage fundamentally requires elastic-wave interactions. However, the practical effectiveness of these active methods for large-scale infrastructure is often constrained by the lack of repeatable and tunable high-energy surface excitation sources, which limit signal stacking, frequency targeting, and quantitative interpretation [17,18,19]. These limitations motivate the development of controllable elastic-wave excitation strategies tailored for robust vertical crack characterization.

Impact sources, such as hammers, steel balls, and spring-loaded impactors, are widely used in elastic-wave-based nondestructive testing because they are low cost, field deployable, and capable of injecting high-energy excitation into attenuative materials, such as concrete [20,21,22,23]. These sources generate short-duration impacts that produce broadband waveforms, which simplifies interpretation by exciting a wide range of frequencies simultaneously. Specifically, impact sources are central to impact-echo testing of plate-like concrete structures, where the dominant frequencies and wave interactions in the broadband response provide information about structural geometry and internal anomalies. However, these sources are not perfectly repeatable or tunable. Impact angle, coupling conditions, and impact velocity affect the force–time history. This changes the bandwidth of the generated waves and alters the wave types dominating the recorded signal [24,25]. This repeatability problem is amplified in concrete with defects (e.g., cracks) because scattering and attenuation complicate both the amplitude stability and the interpretation of wavefields, especially at higher frequencies [26]. As a result, two datasets collected under similar field conditions can still differ enough to mask subtle damage effects, and this uncertainty becomes an obstruction when the goal is to extract quantitative attributes such as phase velocity trends or attenuation parameters rather than relying on a single reflection arrival.

Vertical cracks are a particularly challenging target because their acoustic response depends strongly on crack opening or closure, partial contact between crack faces, and wave polarization and propagation direction. Unlike planar delamination, a vertical crack does not always act as a strong, simple reflector for waves traveling along the surface. Instead, it behaves as a compliant interface that can partially transmit, scatter, and mode-convert the incident wavefield [27,28]. As a result, a crack may be easy to detect from one direction but difficult from another, and its response also depends on frequency because the wavelength relative to crack depth affects how waves scatter and convert [29,30,31]. Crack depth also plays an important role in practice. Shallow cracks may only perturb the near-surface portion of the wavefield, whereas deeper cracks can significantly reduce the effective ligament and produce stronger stiffness contrasts and energy loss [13,28,32]. Despite its practical importance, the influence of crack depth on phase-velocity anisotropy remains insufficiently understood. Clarifying this relationship is critical because deeper cracks pose greater safety risks and can substantially affect infrastructure integrity.

In concrete, high-frequency wave energy attenuates rapidly with propagation distance, creating a tradeoff between resolution, which favors shorter wavelengths, and signal-to-noise ratio. Consequently, stable and repeatable excitation becomes increasingly important to ensure that crack-related responses are not obscured by source variability. The challenges associated with attenuation, wavelength-dependent resolution, and excitation repeatability are well recognized in guided-wave studies. Fundamental Lamb modes, particularly the $A_{0}$ mode in plate-like structures, are commonly used because they concentrate energy near the surface and interact strongly with surface-connected defects [29,33]. In principle, $A_{0}$ dispersion reflects frequency-dependent changes in effective stiffness along the propagation path, while attenuation provides information on scattering and energy loss [34,35]. However, exploiting this sensitivity for crack-depth characterization requires a repeatable excitation source and the ability to control bandwidth adjustment and consistently excite the desired guided-wave mode across different crack depths. To address this limitation, our previous study introduced the “Seesaw Hammer,” a low-cost impact source offering repeatability and tunable frequency bandwidths to enable $A_{0}$ mode excitation in a concrete plate for vertical crack characterization [36]. Using this repeatable source, we showed that fluid in cracks can alter dispersion characteristics of the Lamb wave. Although the Seesaw Hammer demonstrated the utility of mechanical excitation, its reliance on manual actuation still introduced impact variability. This presents a critical fundamental limitation for attenuation analysis. Estimating the quality factor (Q) in highly attenuative media is challenging because it relies on tracking precise spatial amplitude decay. When using conventional manual sources, slight variations in strike angle, impact force, and contact time cause massive shot-to-shot fluctuations in initial wave amplitude and frequency bandwidth. These source-induced variations can mask the true material attenuation, making reliable Q estimation decrease.

The present study addresses this specific knowledge gap through the implementation of a fully automated, computer-controlled linear impact actuator. By establishing closed-loop control over the excitation force and tip mechanics, the automated system locks the input energy and frequency bandwidth in place, eliminating operator-induced variability. This guarantees that any observed amplitude decay is strictly a function of the wave–crack interaction, yielding a stabilized effective attenuation parameter. This highly consistent excitation allows us to quantitatively obtain Q and confidently evaluate its directional anisotropy across vertical cracks of varying depths. In addition, the actuator generates programmable high-energy impacts that improve the signal-to-noise ratio, thereby reducing the need for extensive signal stacking and accelerating data acquisition. The increased impact energy also provides sufficient acoustic energy for characterization of deeper vertical cracks. Furthermore, unlike the mechanically constrained design of the Seesaw Hammer, the actuator enables electronic control of the driving waveform and duty cycle, providing greater flexibility in frequency-content generation and improved dispersion imaging in heterogeneous concrete. The novelty of this study lies not merely in the use of an automated impact source, but in integrating a controllable linear actuator with guided-wave anisotropy and attenuation analysis for quantitative characterization of vertical cracks. Unlike conventional automated impactors designed primarily for repeatability, the proposed framework enables systematic tuning of force history, frequency bandwidth, and excitation conditions while maintaining the shot-to-shot consistency required for robust phase-velocity and Q estimation. Using this system, we evaluate the actuator’s capability for repeatable excitation through variations in impact force, tip stiffness, and tip weight. Additionally, phase velocity is used as the primary metric for quantifying anisotropy, whereas Q is interpreted as an effective modal attenuation parameter. When waves propagate perpendicular to a vertical crack, the incident $A_{0}$ mode crosses a compliant interface and loses coherent energy, while the energy is more preserved when traveling parallel to the crack. If the crack faces are partially closed, opening/closing and frictional sliding may also contribute to energy dissipation, so the effective Q may exhibit direction-dependent behavior, although it is not treated here as a formal anisotropy ratio. Therefore, the reported Q values describe the decay of the coherent $A_{0}$ wave packet, which may include intrinsic absorption, scattering, mode conversion, diffraction, and crack-face interaction. By using this source, we examine how the $A_{0}$ mode’s phase velocity and Q vary with propagation direction relative to a surface-breaking vertical crack and how these responses evolve with crack depth within the same concrete slab.

## 2. Method

### 2.1. Theoretical Background of Lamb Waves

Lamb waves are a type of guided elastic wave that travels in thin, plate-like structures with stress-free surfaces. They arise from a combined behavior of longitudinal and shear-wave components [37]. In theory, Lamb waves result from solving the Navier equations for an isotropic elastic solid while enforcing traction-free boundary conditions at the top and bottom plate surfaces. This is commonly achieved using the Helmholtz decomposition, which separates the displacement field into longitudinal and transverse potentials, leading to the well-known Rayleigh–Lamb transcendental equations. A key outcome of these equations is that Lamb waves are dispersive, meaning their phase velocity varies with the frequency–thickness product. The wavefield is further decomposed into modes based on the symmetry of particle motion about the plate mid-plane: symmetric modes are dominated by in-plane extension/compression, whereas anti-symmetric modes are dominated by out-of-plane flexure (bending) [37]. For a plate thickness of 2*h*, the dispersion relations for symmetric mode and anti-symmetric mode are as follows:

(1) $$\frac{tan(qh)}{tan(ph)}=\;-\frac{4k^{2}qp}{{\left(k^{2}-q^{2}\right)}^{2}}$$

(2) $$\frac{tan(qh)}{tan(ph)}=-\frac{{\left(k^{2}-q^{2}\right)}^{2}}{4k^{2}qp\;}$$

For Equations (1) and (2), k denotes the wavenumber and h is half the plate thickness. We define $p^{2}=\frac{\omega^{2}}{C_{L}^{2}}-k^{2}$ and $q^{2}=\frac{\omega^{2}}{C_{T}^{2}}-k^{2}$, where ω is the angular frequency (ω= 2πf). The material wave speeds are $C_{L}$ (longitudinal wave) and $C_{T}$ (shear wave). Equation (1) corresponds to the symmetric family ($S_{i}$), and Equation (2) corresponds to the anti-symmetric family ($A_{i}$), with i = 0, 1, 2,…. Here we consider the fundamental mode (i = 0). Generally, the $S_{0}$ mode travels faster and is harder to excite than the $A_{0}$ mode. Therefore, we take advantage of this and focus only on the $A_{0}$ mode in this study.

### 2.2. A Linear Impact Actuator as a Controllable Source

**Figure 1:**
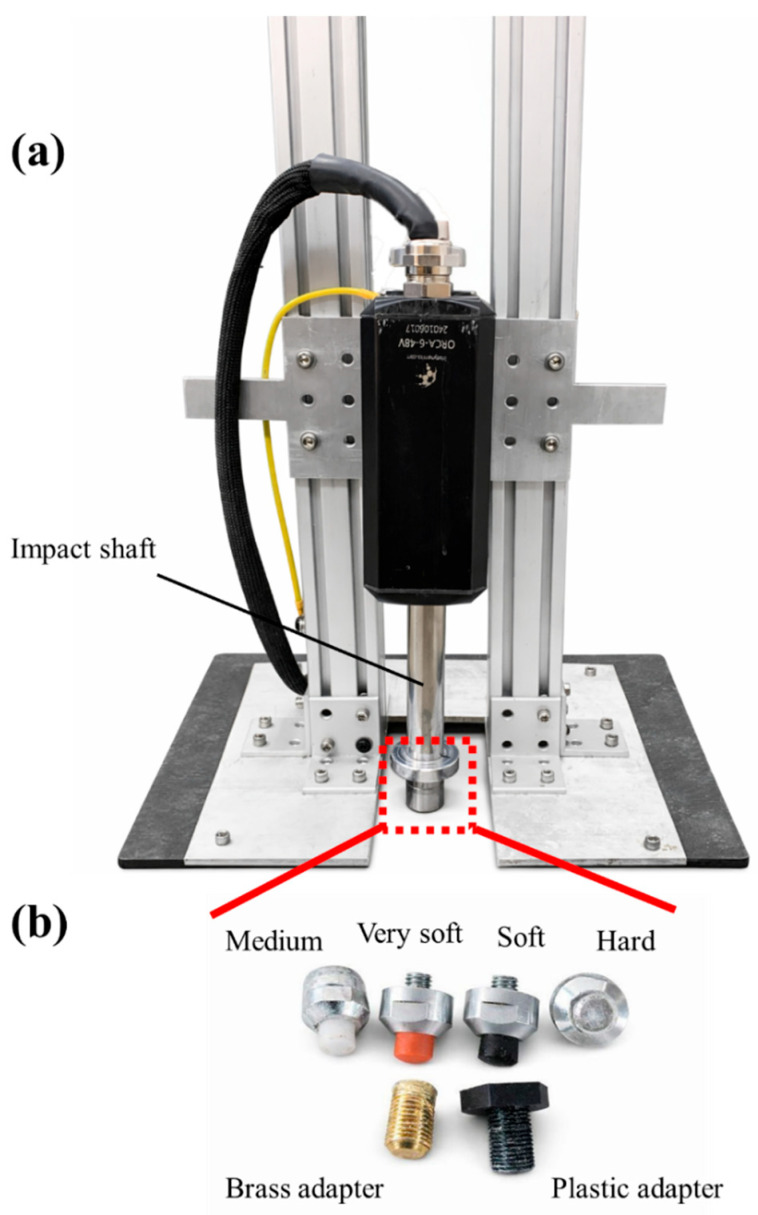
(**a**) A linear impact actuator mounted on an aluminum base, with a black rubber sheet placed between the base and the floor for padding; (**b**) actuator tips with different stiffnesses, along with brass and plastic adapters. To operate the actuator, an adapter is first threaded onto the end of the actuator shaft (marked by the red dashed rectangle), and then the desired tip is threaded into the adapter.

An impact actuator (model ORCA-6-48V by Iris Dynamics Ltd., Victoria, BC, Canada) is linear, offering a compelling solution for generating high-frequency elastic-wave signals (Figure 1a). The actuator’s high-speed and high-force output capabilities make it suitable for generating repeatable, short-duration impacts that are essential for producing broadband acoustic energy in concrete structures. The actuator is a moving-magnet, air-core linear motor in which integrated coils in the stator generate a travelling magnetic field that interacts with permanent magnets embedded in the shaft; an internal controller uses position sensing and closed-loop current control to drive the shaft to commanded forces or positions [38]. In addition to its mechanical capabilities, the actuator is equipped with integrated position and force sensors offering accuracies of ±150 μm and ±0.64 N, respectively [39]. This closed-loop control system facilitates highly repeatable excitation, which is a key requirement for signal stacking and averaging in elastic-wave signal analysis. The actuator’s internal control rate of 3.0 kHz and its compatibility with Modbus RTU and analog/digital I/O interfaces further support precise timing and synchronization with data acquisition systems [39]. These features make it an ideal candidate for automated scanning systems or robotic platforms where consistency and control are critical. Furthermore, we control and configure the actuator motion through IrisControls4 software. The application enables us to monitor the actuator position and impact force, displayed numerically and graphically. Additionally, the software facilitates modification of the operating pulse function (square, sine, sawtooth, and triangle), the amplitude, and the frequency of that pulse. The amplitude expressed in Newtons controls how forceful the shaft strikes are, while the frequency in Hertz controls the rate at which each pulse cycle is repeated. For a square pulse, users may set the duty cycle to display as a percentage.

A linear actuator was mounted vertically in a custom aluminum mount that ensured stable vertical impact motion and minimal lateral motion. The base of the aluminum mount was stabilized during impact using fitness weights (Figure 1a). It is necessary to use an adapter to secure an impact tip that is specifically designed for floor-impact applications. The adaptor can be threaded in through a hole at the tip of the actuator’s shaft, and the impact tip can then be threaded into the adapter. Two adapter types, a lightweight 3D-printed plastic adapter (5 g) and a heavier brass adapter (30 g), were evaluated, and interchangeable hard and medium-hard impact tips from PCB Piezotronics, Depew, NY, USA (model 084B03 and model 084B04, respectively) were evaluated and adopted in this experiment to vary contact stiffness (Figure 1b). To operate the actuator, a power supply (RSP-2000-48) from MEAN WELL USA Inc., Fremont, CA, USA providing 48V DC output was selected to ensure a stable power source for the actuator. The position of the actuator’s shaft was calibrated in the IrisControls4 software before configuring the desired impact motion.

### 2.3. Actuator Characteristics: Repeatability and Tunability

We first verified our actuator by conducting a comprehensive laboratory test. This test examined various impact forces, tip stiffness, adapter type, shot repeatability, waveform functions, and the duty cycle of the square-wave function. Data acquisition was conducted on the concrete floor of the laboratory. The concrete floor, which is approximately 11.43 cm thick, was constructed over a metal deck and reinforced with welded wire fabric. The wires are spaced 6 inches apart in both directions, each with a cross-sectional size of 0.029 square inches. Five R3α accelerometers from Physical Acoustics Corporation, Princeton Junction, NJ, USA selected for their high sensitivity in the 1–30 kHz range and compact footprint, were coupled to the surface of the concrete floor using quick bond gel from the PCB Piezotronics company, Depew, NY, USA (model 080A90). The glue was later removed using an acetone solution. One accelerometer served as the trigger for the acquisition system to begin data collection; it was placed 2 cm away from the impact point of the actuator on the floor. The other four accelerometers served as a signal sensor, with a spacing of 2 cm between each. Coupling quality was verified prior to each acquisition by tapping on the concrete floor and confirming the stability of the recorded impulse response. Signals from the sensors were amplified by 5 V pre-amplifiers from Physical Acoustic, Princeton Junction, NJ, USA and were sampled at 200 kHz using a digitizer from National Instruments corp., Austin, TX, USA (NI PXI-5922). All waveform acquisition, triggering, stacking (signal averaging of repetitive shots), and file management were controlled through a custom LabVIEW 8.6 interface, which ensured consistent timing, automated shot repetition, and direct storage of raw time series. The experiment utilized Multichannel Analysis of Surface Waves (MASW, [40]) principles and a straight-line acquisition geometry. A source offset (the distance between the source and the first sensor) of 5 cm was chosen to optimize excitation of the fundamental antisymmetric ($A_{0}$) mode of the Lamb wave while minimizing higher mode contamination and attenuation of high frequency energy.

To verify the actuator, we first investigated its impact force. A square wave function with a frequency of 3 dHz and a duty cycle of 8% were selected to drive the actuator. These two parameters were fixed, while the amplitude (force) of the square wave function was varied for each dataset: 8 N, 10 N, and 12 N. Each dataset from these forces was obtained using four sensors (four traces). A metal tip with a 30 g brass adapter was used to make an impact, and 25 stacks were performed for each data file.

Furthermore, we tested the repeatable properties of each impact by applying a 10 N square-wave force with the same duty cycle, frequency, and number of stacks. Six different data sets were obtained and analyzed, as presented in the Section 4.

Using the same acquisition parameters, we then collected data for each of the wave functions offered by the actuator to investigate how each function affected the impulse response of the concrete floor. However, in this experiment, a walkaway survey was implemented, in which four fixed accelerometers recorded data while the mechanical impact source, together with a dedicated trigger sensor, was progressively moved to different locations, producing a total of 24 accelerometer signal traces from six shot positions. The larger number of traces allowed us to examine the dispersion properties of the $A_{0}$ Lamb wave mode, as increased spatial sampling facilitates better extraction of Lamb wave information.

While performing this task, we collected data using different duty cycles of the square wave function, specifically 8%, 40%, 72%, and 83% (the maximum at which the actuator’s shaft can operate). An investigation of the tip stiffness of the actuator was also conducted. A metal tip (hard) and a plastic white tip (medium hard) were candidates for this test. We chose 12 N as an impact force so that the medium tip could be triggered properly when hitting the floor, and the other setup parameters remained the same as in the first experiment: four sensors, the square wave, 3 dHz, 8% duty cycle, and 25 stacks. Lastly, we tested different adapter materials and weights by comparing the 30 g brass adapter with the 3D-plastic adapter (5 g), with all other parameters consistent with previous testing.

### 2.4. Experimental Setup and Data Acquisition Procedure

**Figure 2:**
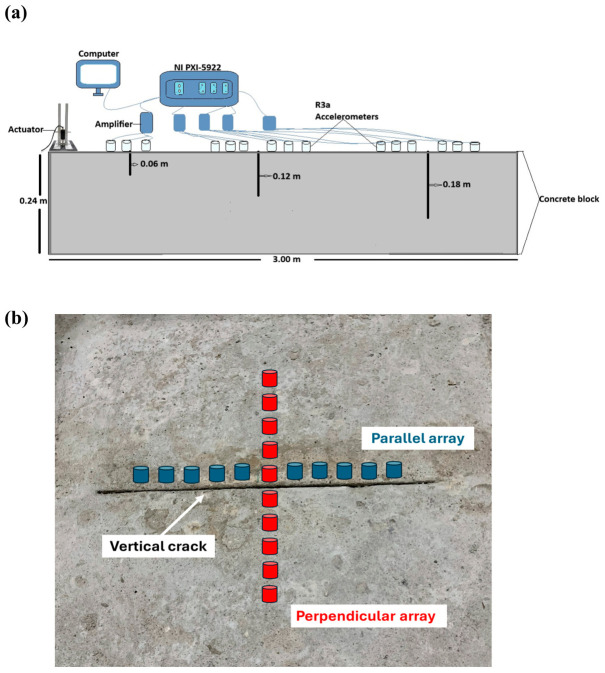
(**a**) Data acquisition system and cross-sectional schematic of the concrete slab. A linear impact actuator generates excitation at the slab surface, while the accelerometers are arranged along the measurement line and connected to an NI PXI-5922 data acquisition system through an amplifier. The locations of vertical surface-breaking cracks at depths of 0.06 m, 0.12 m, and 0.18 m within the 3.0 m long concrete slab are indicated. (**b**) An overhead diagram demonstrates the deployment of accelerometer arrays with respect to the orientation of the vertical crack.

The next step was to apply this source in real case scenarios where vertical cracks were present in a concrete structure. A concrete slab (3.0 m × 3.0 m × 0.24 m) without any reinforcement, acquired from a previous project, was used as an experimental site. An unreinforced concrete slab was intentionally selected for this study to isolate the fundamental wave–crack interactions without the complex scattering and mode conversion effects introduced by reinforcing steel. According to the previous project by Ray et al. [41], the main concrete slab had a density of 2627 kg/m^3^, Young’s modulus of 34.58 GPa, Poisson ratio of 0.2, P-wave velocity of 3824 m/s, and S-wave velocity of 2342 m/s. These parameters were later used for modeling a theoretical dispersion curve, as elaborated in an upcoming section. A 0.1 m thick Styrofoam sheet was placed between the concrete slab and the floor to support the slab and to reduce heat transfer from the floor. The weight of the concrete slab stabilized it on top of the Styrofoam, so no anchors or hooks were necessary to secure the slab to the base floor. We drilled three holes in the middle plane of the slab to depths of 6 cm, 12 cm, and 18 cm, respectively. Next, 20-gauge steel sheets with a thickness of 0.09 cm and a length of 30 cm were placed vertically into each hole, and then the hole was filled in with the same type of concrete material. After allowing the concrete to cure for one week, the removal of the three metal sheets created surface-breaking cracks at three different depths. This method provided a uniform nominal aperture of 0.09 cm, a crack length of 30 cm, and ensured planar, fully separated crack faces, which was necessary to isolate depth-dependent wave scattering from the confounding variables of roughness and partial ligament contact typical of natural cracks. The distance between each crack was 88 cm. This setup is illustrated in Figure 2a.

To estimate optimum acquisition parameters for exciting the $A_{0}$ mode of the Lamb wave and its velocity, a sensor array survey was conducted by collecting data from the intact concrete in the area between the 6 cm crack and the 12 cm crack. Since the square wave performed well in producing $A_{0}$ mode waves for us, we elected to continue with the square wave, using 72% duty cycles and a frequency of 3 dHz. However, the metal tip with the 30 g brass adapter produced wavelengths too short to excite the desired wave properly, so we removed some parts of the adapter to reduce its weight to 20 g and used the white tip with 26 N force to clearly observe $A_{0}$ mode trends. These slab-source settings were selected through expert-guided mode identification of the dispersion images. The selected configuration was required to produce a dominant $A_{0}$ energy ridge that was consistent with the theoretical $A_{0}$ dispersion trend calculated from the slab properties, while reducing obvious higher-mode contamination and maintaining sufficient usable signal energy over the target frequency band. This selection procedure is semi-qualitative and therefore retains some dependence on user experience, as is common in dispersion-curve-based guided-wave analysis. Furthermore, the concrete material was softer than the concrete from the laboratory, so a higher impact force and a lighter weight adapter were needed on the actuator’s tip to properly trigger the acquisition system. A stack number of 25 remained suitable in this environment, where other instrumental noises were sometimes present. The source offset was 5 cm, and the sensor spacing was 2 cm. In this experiment, we kept the source fixed at the same shot location, while sensors were moved progressively to construct a 24-trace virtual record. These acquisition parameters were used for all crack experiments.

We began the experiment with the 6 cm crack. To measure phase-velocity anisotropy, sensor arrays were deployed in two principal orientations, (1) a perpendicular array crossing the crack mid-span and (2) a parallel array aligned along the crack strike, as shown in Figure 2b. This procedure was repeated for all three cracks. For the deepest crack (18 cm), additional data was obtained from perpendicular arrays to evaluate spatial variations in wave speed, attenuation, and anisotropy. Measurements were taken at several key distances from the crack tip: the crack center, 10 cm, 5 cm, the crack tip, and 5 cm past the crack tip.

## 3. Data Processing

Raw voltage amplitudes were exported from LabVIEW as individual .txt files, with each file corresponding to a specific sensor channel. The file was compiled into a single .csv file structured with columns (total traces) and 5000 rows (sample points per trace). The total number of samples collected in this experiment was 5000. A sampling frequency of 200 kHz was used to avoid aliasing. Plots of signal amplitudes (trace versus time) were created by using the CREWES MATLAB toolbox for MATLAB R2024a, which is open for public access [42]. 

A Hanning window filter was applied to the data to suppress spectral leakage, assist in revealing frequency peaks more clearly, and reduce discontinuities at the ends of the trace. Moreover, zero-padding was applied by adding zeros to the end of the sequence until the total length reached 8192 samples. An energy correction factor of 1.6332 was then applied to the output spectrum to compensate for the energy reduction introduced by the Hanning window filter. After obtaining spectra from each trace, we computed the arithmetic mean across all those traces to get a single spectrum for one dataset. Afterwards, a smooth data function with the Gaussian-weighted average method from MATLABtoolboxes was applied to the spectra to smooth out noisy spikes. The spectrum was then normalized by its maximum value.

Finally, the semilogy function was adapted for plotting these spectra in logarithmic scale. This entire process was used for comparing frequency distributions for each impact. Additionally, we plotted frequency trace maps to observe the frequency distribution from every single trace in one dataset. Similar procedures to those mentioned above were applied, except for computing arithmetic means across all traces. The map was plotted using the MATLAB pcolor function, where trace number, frequency, and amplitude spectra are displayed. In addition to looking at amplitude spectra plots, phase velocity and frequency of recorded waves were calculated for a dispersion image. We utilized High Resolution Linear Radon Transform (HRLRT) to transform wavefield data into the phase-velocity-frequency domain. The HRLRT is a powerful elastic-wave processing technique that uses an iterative solver, typically the Conjugate Gradient method, with a specialized pre-weighting scheme to analyze wavefield data. This solver is essentially a repetitive computer process designed to clean up and sharpen the wavefield image by finding the most focused and sparse representation of the signals. Its primary advantage over conventional methods lies in its ability to achieve superior resolution, producing clear images where wave energy peaks are highly focused instead of smeared. This sharpness makes it excellent at separating closely spaced wave types (different surface wave modes) and gives it enhanced resistance to random noise and poor data quality. Furthermore, the HRLRT is robust enough to provide reliable results even when measurement points are irregularly spaced or the data collection line is short [43]. Before running the transformation, we applied a cosine taper window to the data to isolate reflection noises from the true events. As a result, the final dispersion image dominantly displayed energy from the interested wavelets, making the $A_{0}$ trend clearer. However, even without a window applied to the data, the final images were still clear enough for extracting dispersion curves.

Once meaningful dispersion images exhibiting clear $A_{0}$-mode trends were obtained for each dataset, the $A_{0}$ dispersion curves were extracted from the two-dimensional frequency–velocity plots. A polygonal region of interest (ROI) was manually defined using the MATLAB function roipoly to isolate the high-energy region associated with the $A_{0}$ mode while excluding dominant artifacts and higher-order modes. Although the ROI was selected manually, it served mainly as a physical bounding constraint for the subsequent automated extraction. Within the selected ROI, the dispersion trend was algorithmically tracked by identifying the local energy maxima at each frequency bin, ensuring that the extracted phase-velocity curve remained data-driven. The extracted frequency–velocity pairs were subsequently fitted using the MATLAB Curve Fitting Toolbox (cftool) with a Least Absolute Residuals (LAR) regression algorithm (L1-norm). Compared with conventional least-squares fitting, LAR is less sensitive to outliers and noisy data, allowing the fitted curve to more accurately represent the dominant energy ridge of the $A_{0}$ mode while reducing the influence of localized disturbances caused by scattering. In regions where scattering or material heterogeneity fragments the energy ridge, the resulting increase in dispersion is reflected by wider stability bounds, thereby quantifying the uncertainty associated with the extracted dispersion curve. The final extracted curve was overlaid on the dispersion image and compared with the theoretical $A_{0}$-mode dispersion curve calculated by solving Equation (2). To assess the reliability of the final extracted curves, we computed statistical stability bounds. We emphasize that these bounds serve as an independent measure of uncertainty for each frequency bin, rather than a constraint to guide the initial curve extraction. During this evaluation, the algorithm applies an 800 m/s velocity window (±400 m/s) centered on the extracted dispersion curve to separate relevant signals from background artifacts. Inside this band, the dispersion image’s normalized energy intensities act as statistical weights, meaning peak intensities (dark red on the color scale) receive maximum weight, while low-level noise (dark blue) receives minimum weight. These weights allow us to calculate both a weighted mean velocity and a weighted standard deviation (*σ*). The resulting standard deviations dictate the upper and lower stability bound relative to the mean. If dispersion energy is highly diffuse or noisy, the resulting standard deviations consequently expand, yielding wider stability bounds. This 800 m/s window is wide enough to prevent the algorithm from tracking the wrong ridge. If the algorithm accidentally captures a noise peak, the high intensity of the true $A_{0}$ energy ridge will remain inside this window and get assigned more weight, which increases the standard deviation and exposes the error. Larger velocity windows can inflate the estimated uncertainty by including higher-order modes and random noise; therefore, we used a constant window of ±400 m/s throughout the stability analysis. The stability bounds are defined as the weighted mean velocity *± σ*.

The next data-processing step involved calculating the quality factor (Q), which characterizes the energy dissipation capabilities of concrete materials. Q is dimensionless and represents intrinsic attenuation, the irreversible transformation of elastic-wave energy into heat. While other factors, such as geometrical spreading, scattering, and multipathing, also contribute to the reduction in wave amplitude over distance, intrinsic attenuation is a material-specific property. In this study, the estimation of wave attenuation relies on the amplitude decay model described by Stein and Wysession [44]:

(3) $$A(x)\;=\;A_{0}e^{\left(-\frac{\omega x}{2cQ}\right)}$$

where A(x) denotes wave amplitude (V) in the time domain at a distance x(m), $A_{0}$ denotes an initial wave amplitude (V) in the time domain at an excitation location, ω denotes an angular frequency of a wave and equals 2πf, where f is the central frequency of the waves (Hz), c denotes a phase velocity (m/s), and Q denotes a quality factor (dimensionless). Since the traveling waves themselves also lose their amplitudes from their front spreading, we accounted for this factor by multiplying Equation (3) by $\frac{1}{x^{\alpha }}$, where α denotes a geometrical spreading power. We then arranged the equation for the simplicity of Q calculation:

(4) $$A(x)\;=\;\frac{1}{x^{\alpha }}{Ce}^{\left(-\beta x\right)}$$

where C represents $A_{0}$, and β represents $\frac{\omega }{2cQ}$. For the entire length of data records, we extracted the absolute maximum amplitude from each trace (24 values for 24 traces in one dataset) by using the max function in MATLAB; each amplitude point corresponds to its sensor distance from the source. Before the extraction, we ensured that the data was not contaminated by noise or boundary reflections by comparing the amplitude spectrum of the main events with that of the noise. Additionally, though this actuator could produce broadband frequency up to 20 kHz, the dominant frequencies stayed in a range of 300–2000 Hz. In this range, the wavelengths were relatively long compared to the crack depths, and therefore they were not effective in characterizing the cracks. Thus, we applied the Parks–McClellan filter to remove this frequency range with a lower stopband edge at 2 kHz, a higher stopband edge at 20 kHz, a transition band of 500 Hz, and a filter order of 3000. The Parks–McClellan is a finite impulse response (FIR) filter with an equiripple frequency response and sharp transition bands, providing stable filtering of noisy data [45]. After filtering, we extracted the absolute maximum amplitude from the filtered time-series by using the max function. The dispersion analysis ensures that the acoustic energy within this band is dominated by the $A_{0}$ Lamb mode and we consistently tracked the peak envelope of the $A_{0}$ modal packet, rather than capturing arbitrary background noise or non-propagating scattered energy. To calculate Q, we adopted the Bootstrap method, which quantifies the uncertainties in parameter fitting for Q,α,C, and β [46]. Generally, this method resamples the input amplitude points with replacement into new datasets (1000 sets in this study). Each set is used for fitting using Equation (4), and the uncertainty for each parameter is finally determined from the distribution of these 1000 fitting results. From valid fitting results (excluding iterations where parameters hit the fitting bounds or the estimated Q exceeds 1000), we calculated the mean value and the 95% confidence interval for each parameter from the fitting distribution. The central frequency was taken directly from the filtered data, whereas the velocity was extracted from the phase velocity curve, and Q was calculated using the associated velocity and central frequency. To run Bootstrap, we were required to estimate the fitting bounds for α, β, and C to limit the searching boundary. Theoretically, in an isotropic and homogeneous medium, body and surface waves have geometrical spreading powers of 1.0 and 0.5, respectively. However, these values deviate when the wavefield interacts with a highly heterogeneous medium or macroscopic defects. For $A_{0}$ mode waves, we set this bound to (0.1 and 3.0) to accommodate the severe geometric scattering and spatial wavefield distortion induced by the cracks and heterogeneity in concrete. It should be emphasized that the geometrical spreading term used in Equation (4) is an empirical approximation. Lamb-wave spreading in a finite plate is more complex than the idealized spreading of body waves because the wavefield is guided by the plate boundaries and may be affected by near-field effects, multimodal interference, scattering from aggregates, and scattering or mode conversion at the crack. Therefore, a single spreading exponent cannot fully represent the true distance- and frequency-dependent spreading behavior of Lamb waves. In this study, α is treated as an effective spreading parameter that absorbs the combined influence of geometrical spreading, waveguide behavior, and crack-induced wavefield redistribution over the measured propagation path. For β, which is inversely related to Q in terms of $\frac{\omega }{2cQ}$, we expect Q in concrete to be in a range of 0.5 to 50, according to Rhazi and Kodjo in [47], who previously determined Q in concrete materials. We applied this range, along with the corresponding velocity and frequency, to obtain the β range needed for Bootstrap. For C, which represents the initial wave amplitude at the source location, we used the amplitude value from the sensor closest to the source. It was constrained within one order of magnitude of the maximum observed amplitude (0.1$A_{max}$ and 10.0$A_{max}$) to ensure numerical stability and prevent the nonlinear optimizer from converging to unrealistic source strengths. Before the final analysis, outlier points that did not follow the expected physical trend were removed. This was completed by temporarily straightening the curved amplitude data into a linear trend; any points that strayed significantly from this line (more than 1.5–2.0 standard deviations) were removed to prevent experimental errors from affecting the results. Hence, the removal step assures that the subsequent Bootstrap analysis accurately represents the true uncertainty of the physical wavefield attenuation rather than inflating the error bounds with localized sensor debonding.

The last step in data processing involved an estimation of the anisotropy ratio to quantify the severity of the vertical cracks. Following Jeng et al. [48], who calculated seismic radial anisotropy by utilizing the difference between vertically and horizontally polarized shear wave velocities, we adapted their methodology for the Lamb wave traveling through the vertical cracks in a concrete slab by replacing the shear velocity components with the phase velocity of the $A_{0}$ mode from both parallel and perpendicular configurations. We defined the anisotropy ratio (η) as:

(5) $$\eta =\frac{V_{Parallel}-\;V_{Perpendicular}}{V_{iso}}$$

where $V_{Parallel}$ and $V_{Perpendicular}$ denote a frequency-dependent phase velocity of the $A_{0}$ mode when the sensor array is parallel and perpendicular to the crack plane, respectively. $V_{iso}$ is an effective isotropic velocity analogous to the isotropic component used in seismic radial anisotropy studies calculated as:

(6) $$V_{iso}=\sqrt{\frac{2V_{Perpendicular}^{2}+V_{Parallel}^{2}}{3}}$$

Since the velocity is frequency dependent, we extracted the velocity–frequency pair from a dispersion curve and used it to estimate η. The calculation results are discussed in the following section.

## 4. Results

### 4.1. Performance Validation of the Impact Actuator

**Figure 3:**
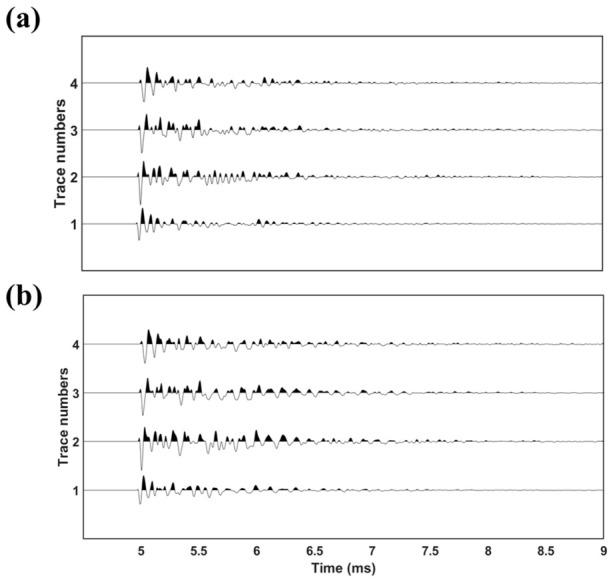
(**a**) Sensor responses obtained with an 8 N impact using a metal tip, a 30 g brass adapter, and a square-wave driving signal (3 dHz, 8% duty cycle). (**b**) Sensor responses obtained with a 10 N impact under the same experimental conditions as in (**a**). The trace numbers correspond to the sensor numbers. Positive signal amplitudes are shaded black for visualization.

**Figure 4:**
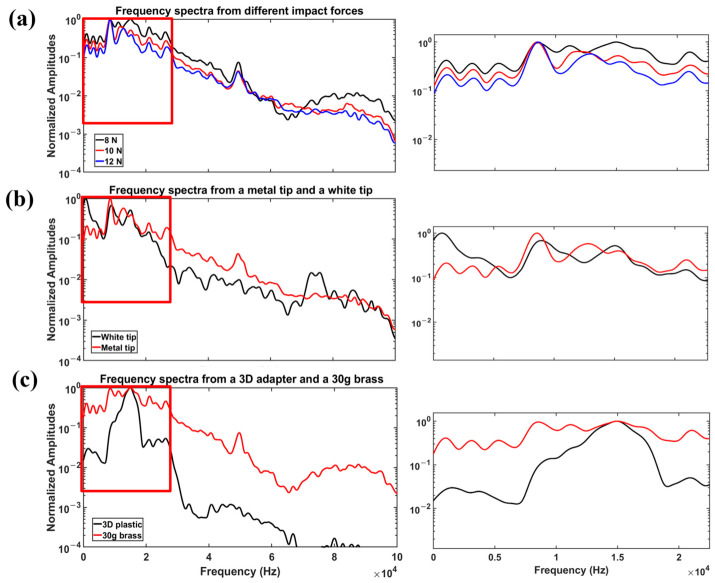
(**a**) Average frequency spectra calculated from the datasets corresponding to 8 N, 10 N, and 12 N impacts. (**b**) Average frequency spectra obtained using a white tip and a metal tip with a 30 g brass adapter, a 12 N impact, and the same square-wave signal as in (**a**). (**c**) Average spectra obtained using a 3D-printed plastic adapter and a 30 g brass adapter with an 8 N impact and the same driving signal. The red boxes in the left panels indicate the regions that are magnified in the corresponding right panels.

**Figure 5:**
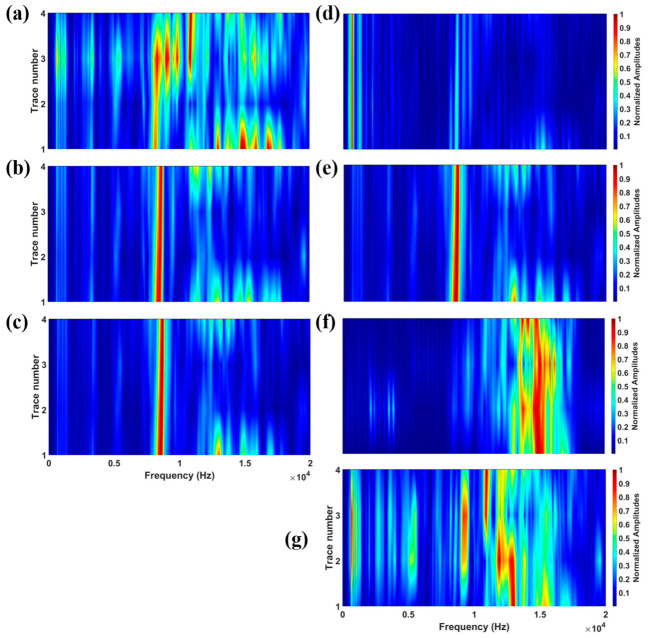
(**a**–**c**) Frequency–trace maps corresponding to Figure 4a, with the top, middle, and bottom panels representing the 8 N, 10 N, and 12 N impacts, respectively. (**d**,**e**) Frequency–trace maps corresponding to Figure 4b, where the top panel shows the white tip and the bottom panel shows the metal tip. (**f**,**g**) Frequency–trace maps corresponding to Figure 4c, where the top panel shows the 3D-printed plastic adapter and the bottom panel shows the 30 g brass adapter. Each trace number indicates the corresponding sensor, and the lower color-bar threshold (0.1) is selected for visualization purposes to improve contrast of the dominant spectral features and suppress low-amplitude background noises.

In this section, we discuss comprehensive laboratory testing of the actuator’s performance. The tests described in Section 2 include investigations of different impact forces, adapters, tip materials, input waveforms, and the repeatability of single impacts. Because the actuator allows the user to vary the impact force, we examine how the frequency spectra change with different hitting strengths. First, Figure 3a,b illustrate raw signal data that we collected from the laboratory’s concrete floor without any filter applied. The *y*-axis represents trace numbers or sensor numbers, and the *x*-axis represents time in ms. In the signal records, we observed only the Lamb wave event because the concrete floor is thin, and only the vertical receivers are used. The amplitude frequency spectrum from different forces is displayed in Figure 4a. Since each spectrum is normalized by its maximum value, the amplitudes imply the relative frequency contents. The amplitude spectrum corresponding to the 8 N impact (black curve) exhibits relatively higher frequency content than that of the 10 N and 12 N impacts. However, without normalization, the order of these spectra is reversed, with the 12 N impact producing the highest amplitude. Figure 5a–c present frequency–trace maps for the three impact forces: 8 N (top), 10 N (middle), and 12 N (bottom). These maps display the spectra for each sensor response, enabling visualization of the frequency distribution across the entire sensor array. After normalization, the 8 N map shows stronger high-frequency intensity (dark red) up to approximately 20 kHz, followed by the 10 N and 12 N maps. This observation is consistent with the result shown in Figure 4a.

In addition, mechanical excitations were performed using different tip materials to investigate how the generated frequency spectra depend on tip stiffness. The spectral results for the white tip and the metal tip are shown in Figure 4b. The same procedure used to obtain the average spectra was applied. In the lower-frequency regime (up to approximately 7 kHz), the spectrum generated by the white tip dominates but gradually decreases beyond this range, after which the metal-tip spectrum becomes dominant. In general, the metal tip also produces stronger signals than the white tip, as evidenced by the clear separation between their un-normalized spectra. The frequency–trace maps further support this observation. Between 7 kHz and 20 kHz, the metal-tip map shows widespread orange and red regions, indicating higher spectral amplitudes.

In addition to testing different tip materials, the adapter weight and material were also evaluated. Using the same metal tip, the spectrum generated with the 30 g adapter is clearly stronger and more broadband than that produced by the 5 g plastic adapter, suggesting that it is more suitable for exploration applications, as investigated further in this study. Interestingly, the plastic adapter produces a narrower spectral band, which may be advantageous for other research applications. The corresponding results are shown in Figure 4c and Figure 5f,g.

**Figure 6:**
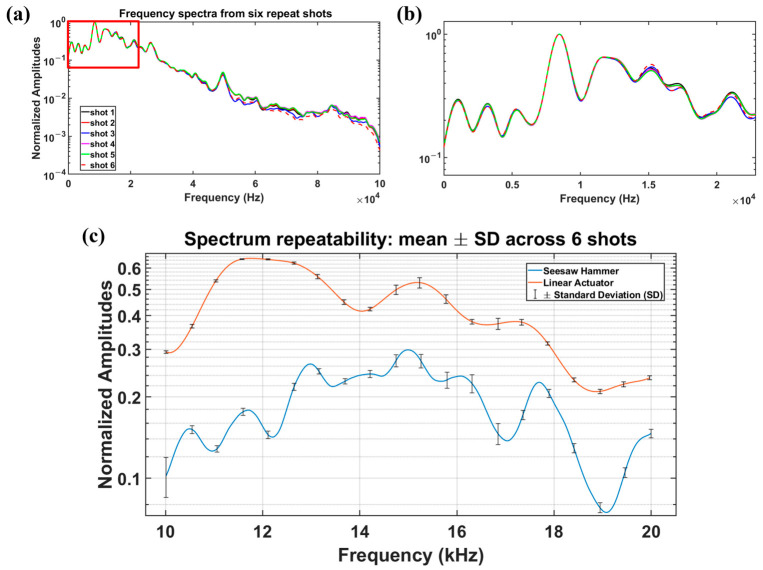
(**a**) Average frequency spectra from six repeated shots produced by the square wave function of 10 N with a frequency of 3 dHz and a duty of 8%; (**b**) a zoom-in window of the region indicated by the red box in (**a**). (**c**) A quantitative analysis of (**a**) plotted in orange with repeatability test result from the Seesaw Hammer plotted in blue, where a mean spectrum from all shots is calculated and plotted with error bars as standard deviation (SD), with the Seesaw Hammer data adapted from the open dataset in [36].

To evaluate the repeatability of this tunable source, we examined the average spectra from six identical shots. Figure 6 summarizes the shot-to-shot consistency of the actuator under identical driving conditions. The spectra from the six shots are compared qualitatively and show strong overlap in amplitude up to approximately 40 kHz, after which some variability appears toward the Nyquist frequency (100 kHz). The zoomed-in view in Figure 6b highlights a stable low-frequency signature, with a dominant peak around 8 kHz and consistent secondary peaks in the 10–20 kHz range.

This repeatability is further quantified in Figure 6c, where the mean spectrum over the 10–20 kHz band is shown with ±1 SD error bars (*n* = 6). The small scatter relative to the mean confirms minimal spectral variability between shots and supports the reliability of the source for repeatable broadband excitation. We further compared the repeatability of the impact actuator with that of the Seesaw Hammer. The spectrum in blue presents the repeatability behavior of the previously developed Seesaw Hammer source reported by Srisapan et al. [36]. According to Figure 6c, the linear impact actuator produces a smoother broadband excitation, dominated by a spectral peak near 11.5–12.0 kHz, whereas the Seesaw Hammer exhibits a more erratic response with its primary energy concentrated near 15.0 kHz. In addition, the impact actuator demonstrates smaller spectral scatter relative to the mean response over the 10–20 kHz band, indicating improved shot-to-shot consistency. For a more quantitative comparison, we calculated the coefficient of variation (CV), defined as

(7) $$CV(f)=\frac{SD(f)}{\mu (f)}$$

where μ and SD are the mean and standard deviation of the amplitude spectrum at frequency (f), respectively. We used the CV instead of the standard deviation because it normalizes variability by signal amplitude, enabling a fair comparison of repeatability between the two sources despite differences in signal strength. This normalization is particularly important because the impact actuator generates stronger signals and therefore naturally produces larger absolute spectral amplitudes. The average CV of the impact actuator was 0.0199, whereas the Seesaw Hammer exhibited an average CV of 0.0488. These results indicate that the Seesaw Hammer produces approximately 2.45 times greater relative variability than the linear impact actuator. Equivalently, the proposed source reduces relative shot-to-shot variability by approximately 60%, demonstrating substantially improved spectral repeatability.

**Figure 7:**
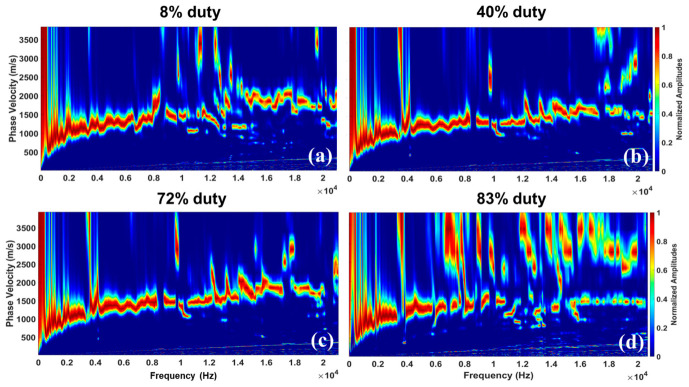
Dispersion images obtained from the metal tip, the 30 g brass adapter, and the 10 N of the square wave function with 3 dHz and different duty cycles: (**a**) 8%, (**b**) 40%, (**c**) 72%, and (**d**) 83%. For optimal visualization, the lower bound of the color scale is set to 0.1 to mask background noise and clearly isolate the dominant spectral energy.

The last parameter we investigated is the driving waveform (sine, square, triangle, and sawtooth). Because the actuator shaft response is controlled by the waveform shape, different waveforms create different shaft acceleration and contact behavior. Sine and square waves tend to produce more stable contact timing, which results in a more consistent impact strength. In contrast, triangle and sawtooth waveforms spend less time at high command levels, which can reduce the effective contact impulse and lead to a weaker impact. Figure 7 shows how to change only the duty cycle while keeping the metal tip, 30 g brass adapter, 10 N amplitude, and 3 dHz square-wave input the same affects the resulting dispersion images. It should be noted that these images are generated from a 24-sensor array, which facilitates the calculation algorithm. In all four images, a continuous high-amplitude ridge is still observed, suggesting that the main guided-wave dispersion trend is generally preserved regardless of duty cycle. However, the image quality and energy distribution vary noticeably. For duty cycles of 40% and 72%, the dominant ridge appears sharper and more continuous across frequency, making it easier to follow. For duty cycles of 8% and 83%, additional vertical streaks and scattered energy bands become more pronounced, which reduces the contrast of the main ridge and makes the dispersion pattern less clean. Overall, these observations suggest that duty cycle also influences how clearly the dispersion images are expressed, which directly relates to how the actuator’s shaft functions through the driving command. This observation led us to use the square wave with 72% duty cycle to further investigate the crack in concrete.

### 4.2. Implementation of the Impact Actuator on a Concrete Slab with Vertical Cracks

In this section, we discuss the experimental results on the surface-breaking vertical cracks embedded in the concrete slab at different depths by using the impact actuator as the elastic-wave source. The experiments focus on the analysis of the phase-velocity anisotropy for each crack depth along with their quality factor.

#### 4.2.1. Phase-Velocity Anisotropy

At the beginning of the experiments, our task for obtaining meaningful data was to customize the actuator’s parameters to correlate with a new experimental site. Since the concrete slab is more than twice the thickness of the slab in the laboratory, the Lamb wave’s $A_{0}$ mode becomes obscured when using the metal tip and the 30 g adapter. We solved this by reducing the weight of the adapter (now the 20 g brass), using the medium white tip, and increasing the impact force of the actuator to 26 N. With these settings, the wavelengths of the wave become comparable to or greater than the thickness of the medium, allowing proper excitation of the Lamb wave. The source setting procedures and selection criteria of coherent $A_{0}$ energy ridge are already discussed in Section 2.4.

**Figure 8:**
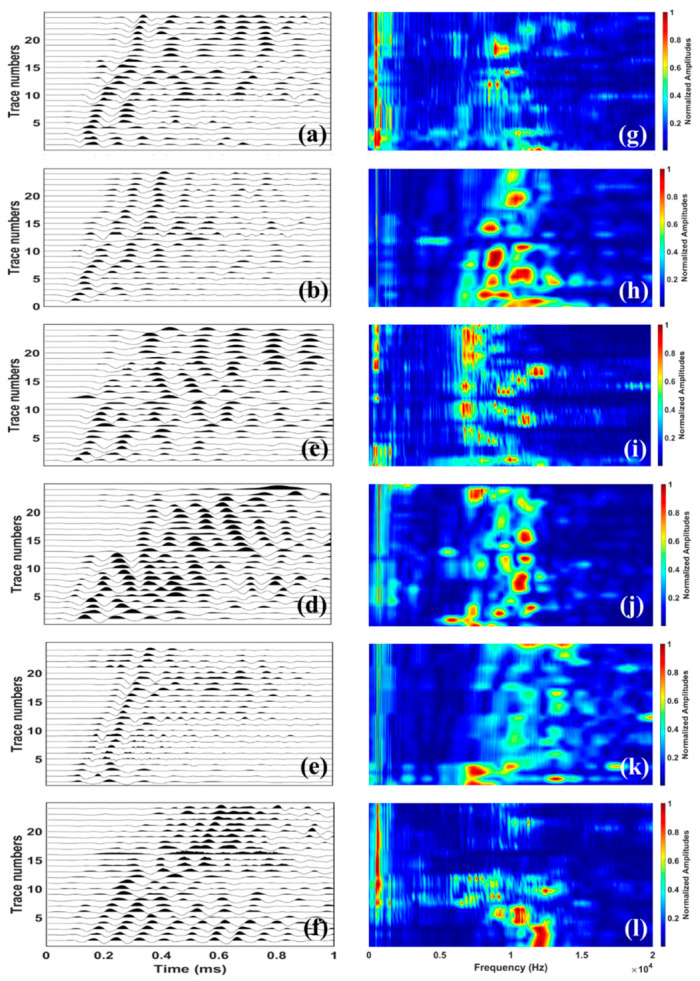
Sensor responses from cracks at three depths. Panels (**a**,**b**) show the 6 cm crack for the parallel and perpendicular arrays, respectively. Panels (**c**,**d**) show the 12 cm crack, and panels (**e**,**f**) show the 18 cm crack, again for the parallel and perpendicular arrays. Panels (**g**,**h**) present the corresponding frequency–trace maps for (**a**,**b**), panels (**i**,**j**) for (**c**,**d**), and panels (**k**,**l**) for (**e**,**f**). Note that trace numbers denote sensor numbers and a visual threshold of 0.1 is applied to the normalized amplitude scale in color bar for better visualization.

Figure 8 provides an overview of the wavefield characteristics for the three crack depths and illustrates how the response changes with array orientation. In all cases, a coherent guided-wave packet arrives within the first millisecond; however, the waveform characteristics vary with both crack depth and array orientation. For the 6 cm crack, the parallel array configuration in Figure 8a exhibits cleaner and more consistent waveforms across traces, whereas the perpendicular configuration in Figure 8b shows greater amplitude variability and slightly more irregular wave-train shapes. The frequency–trace maps show a similar trend: Figure 8g displays more diffuse and weaker energy, while Figure 8h contains several stronger and more localized high-frequency regions.

When the crack depth increases to 12 cm, the records in Figure 8c,d become noticeably more complex, with stronger interference patterns and increased trace-to-trace variability. This behavior is reflected in Figure 8i,j, where the spectral energy is no longer broadly distributed but instead appears in multiple peaks within the dominant frequency band, suggesting increased scattering and mode mixing.

For the deepest crack (18 cm), the contrast between array orientations becomes even more pronounced. The parallel configuration in Figure 8e shows strong energy confined to fewer traces, whereas the perpendicular configuration in Figure 8f exhibits high reflection and a scattering event. The corresponding frequency–trace maps further support this observation. Figure 8k shows predominantly low-level background energy with only small, localized regions of higher amplitude, while Figure 8l exhibits more distinct high-amplitude regions.

Overall, Figure 8 demonstrates that both crack depth and array orientation leave clear signatures in the data, not only in the waveform characteristics but also in the distribution of spectral energy across the receiver array.

**Figure 9:**
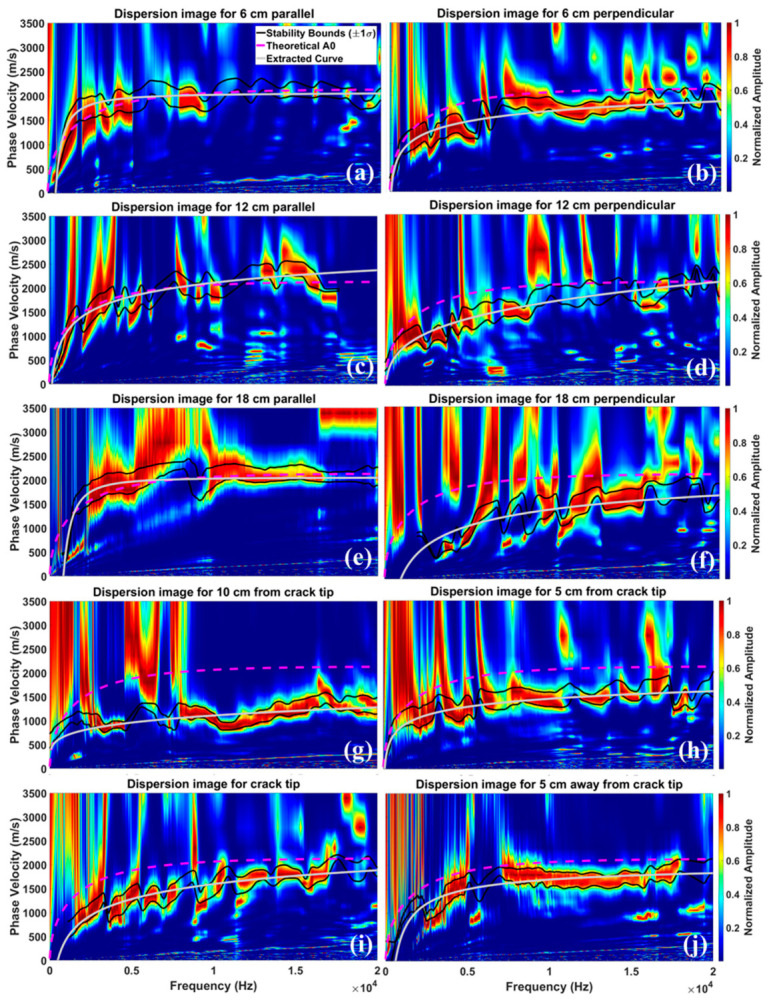
Dispersion images obtained from HRLRT, shown with the extracted phase-velocity curve (solid gray), the theoretical curve (dash pink), and the stability bounds (solid black). The stability bounds represent the weighted standard deviation (±1σ) of the dispersion intensity within a ±400 m/s window. Panels (**a**,**b**) correspond to the 6 cm crack, (**c**,**d**) to the 12 cm crack, and (**e**,**f**) to the 18 cm crack. Panels (**g**–**j**) show the lateral variation for the 18 cm crack. The lower bound of the color bar represents the visualization threshold of 0.1 (10% of the normalized peak) to enhance dominant spectral energy.

Figure 9 presents the HRLRT dispersion images together with the extracted phase-velocity curves, the theoretical $A_{0}$ trend, and the stability bounds. Overall, the $A_{0}$ branch appears as the dominant high-energy ridge across the 0–20 kHz band; however, its clarity and velocity position depend strongly on both crack depth and array orientation.

For the 6 cm crack, the parallel configuration in Figure 9a produces a relatively clean and continuous ridge that approaches the expected $A_{0}$ velocity, and the extracted curve closely follows the theoretical trend in the mid-frequency range. In contrast, the perpendicular configuration in Figure 9b still shows the characteristic $A_{0}$ curvature, but the energy ridge is more fragmented, and the extracted curve tends to shift toward lower velocities, suggesting stronger scattering and possible mode mixing in this geometry.

For the 12 cm crack (Figure 9c,d), the $A_{0}$ ridge remains identifiable, although the dispersion images become more complex, with additional peaks and vertical streaks. The extracted curves still track the dominant ridge, but the agreement with the theoretical $A_{0}$ trend decreases, particularly at higher frequencies where the spectral energy becomes more discontinuous.

For the deepest crack (18 cm), the difference between array orientations becomes more pronounced. The parallel configuration in Figure 9e produces the strongest and most continuous $A_{0}$ ridge, and the extracted curve remains well aligned with this ridge over a broad frequency range. In contrast, the perpendicular configuration in Figure 9f is dominated by irregular energy columns and a lower-velocity ridge, causing the extracted curve to deviate further from the theoretical $A_{0}$ trend.

Figure 9g–j further demonstrate that the 18 cm case exhibits lateral variability. As the observation position shifts relative to the crack tip, the dominant ridge changes noticeably, and the extracted curve varies accordingly, indicating that the local wavefield around the crack is not uniform along its length.

The stability bounds quantify how tightly the dispersion energy is concentrated around the extracted ridge within the velocity window. Narrow bounds indicate a sharp, well-defined ridge and therefore high confidence in the extracted phase-velocity curve. Wider bounds reflect multi-peaked ridges and are consistent with mode interference, scattering, or reduced signal coherence. These regions correspond to lower-confidence portions of the extracted curve and should therefore be interpreted with caution. Nevertheless, the stability bounds remain relatively tight over most frequency ranges where the $A_{0}$ energy ridge is continuous.

**Figure 10:**
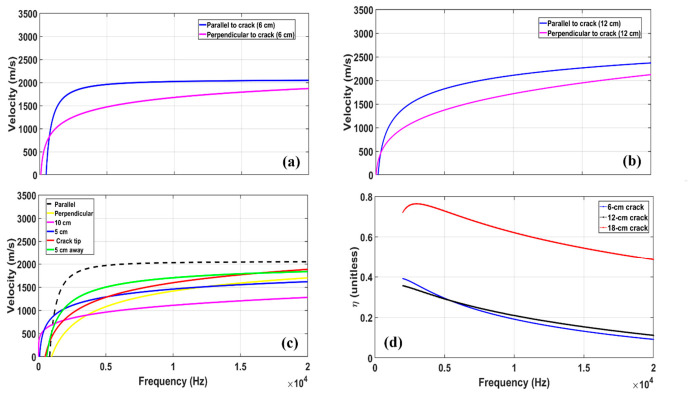
Extracted dispersion curves from Figure 9. Panel (**a**) corresponds to the 6 cm crack, (**b**) to the 12 cm crack, and (**c**) to the 18 cm crack, and (**d**) displays the anisotropy ratio (*η*) from all cracks for each frequency.

Figure 10 summarizes the HRLRT-extracted phase-velocity curves to facilitate direct comparison between array orientations and crack depths. For the 6 cm and 12 cm cracks in Figure 10a,b, a consistent pattern emerges: the curve obtained from the parallel array configuration remains systematically higher than that from the perpendicular configuration across the entire frequency band (up to 20 kHz). This separation is already visible at low frequencies and persists as the curves gradually approach their high-frequency asymptotic behavior.

For the 18 cm crack in Figure 10c, the orientation effect becomes even more pronounced. In addition to the differences between the parallel and perpendicular configurations, the curves extracted at different lateral positions exhibit noticeable shifts in both velocity magnitude and curvature, even though they correspond to the same dominant $A_{0}$ ridge. This behavior indicates that the dispersion response is not uniform along the crack length.

Figure 10d summarizes this behavior using the anisotropy parameter η, calculated from Equation (5) for each frequency index. The 18 cm crack exhibits the strongest anisotropy across the frequency band, whereas the 6 cm and 12 cm cracks show lower values and follow a similar decreasing trend with increasing frequency. In other words, as crack depth increases, the difference between the parallel and perpendicular configurations becomes more pronounced and persists across a wide frequency range. For the deepest crack, this anisotropic response also varies with the lateral position of the array relative to the crack tip.

#### 4.2.2. Quality Factor

**Figure 11:**
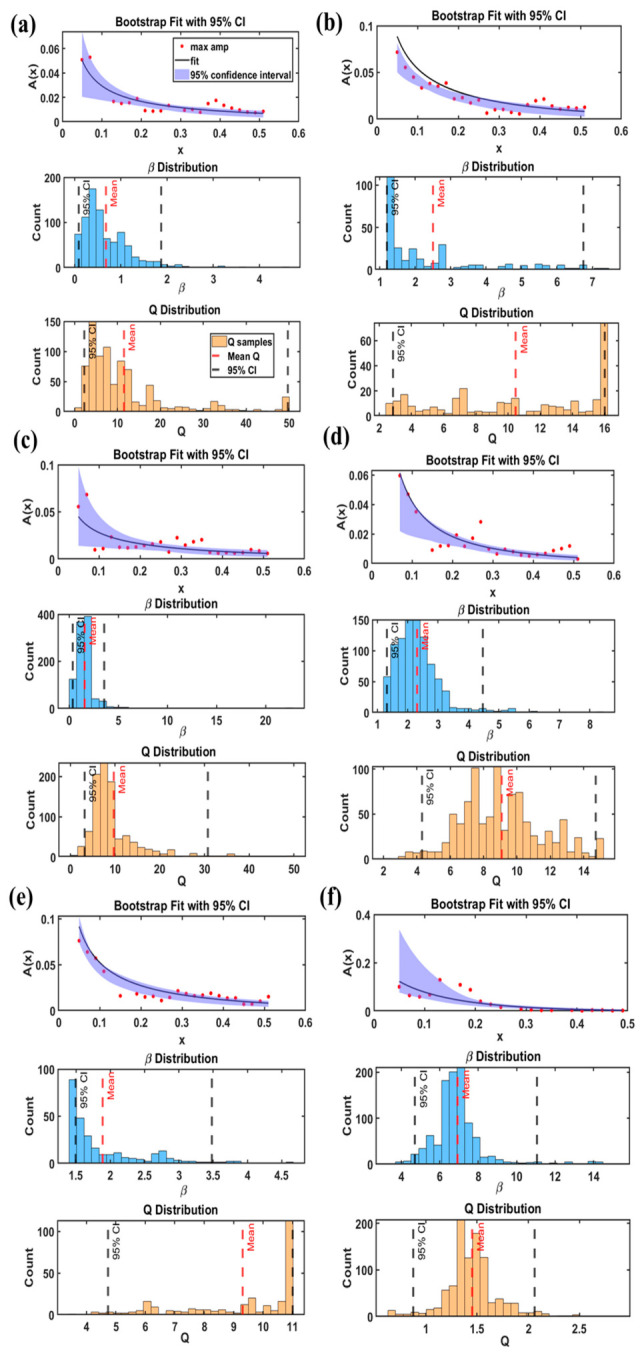
Bootstrap results for quality factor (Q) estimation. For each case, the top panel shows the Bootstrap fit of the amplitude–distance decay A(x) with the 95% confidence band, while the middle and bottom panels show the corresponding Bootstrap distributions of *β* and Q, respectively, with the mean and 95% confidence intervals indicated. Panels (**a**,**b**) correspond to the 6 cm crack for the parallel and perpendicular array orientations, respectively; (**c**,**d**) correspond to the 12 cm crack; and (**e**,**f**) correspond to the 18 cm crack.

In this section, we present Q estimation results obtained using the Bootstrap approach. Table 1 lists the parameters used to calculate Q according to Equation (4). Figure 11 shows that the amplitude–distance decay can be fitted consistently in all cases; however, the stability of the fit and its implications for attenuation vary with crack depth and array orientation.

For the 6 cm crack (Figure 11a,b), the Bootstrap fits capture the overall decay trend, although the uncertainty is larger at short offsets where several data points deviate from the main trend. This variability is reflected in the histograms, where the β and Q distributions appear broader and slightly skewed, indicating that the estimated attenuation is sensitive to trace selection during Bootstrap resampling.

For the 12 cm crack (Figure 11c,d), the decay fits remain smooth, and the β distributions become narrower compared with the 6 cm case. Consequently, the Q estimates cluster more tightly around a mid-range value with a reduced confidence interval, indicating improved stability of the attenuation estimates.

The strongest contrast occurs for the 18 cm crack (Figure 11e,f). In the parallel orientation, the Q estimates shift toward higher values, with the histogram accumulating near the upper bound of the tested range, suggesting relatively weak attenuation. In contrast, the perpendicular orientation exhibits larger β values and a tightly clustered low-Q distribution, consistent with a much steeper amplitude decay and stronger energy loss along this measurement direction.

Overall, the Bootstrap analysis supports the observations from the waveform and dispersion analyses. Although crack depth influences attenuation behavior, array orientation exerts an even stronger effect, particularly for the deepest crack. In the parallel configuration (Figure 11a,c,e), the fitted amplitude–distance decay changes only slightly, from 6 to 18 cm, and the β and Q distributions remain within a similar range. In contrast, the perpendicular configuration (Figure 11b,d,f) exhibits progressively stronger attenuation with increasing crack depth. Specifically, β increases and Q decreases from the 6 cm to the 12 cm case, with the 18 cm crack showing the steepest decay and the most tightly clustered low-Q distribution.

It should be noted that some scatter in the amplitude measurements may result from weak or variable coupling; therefore, the reported values represent effective Q estimates, with uncertainty captured by the Bootstrap confidence intervals. Additional Bootstrap results for lateral measurements of the 18 cm crack are provided in the Supplementary Materials.

## 5. Discussion

### 5.1. The Impact of the Actuator’s Performance and Controllability

Laboratory validation confirms that the impact actuator can deliver short, repeatable impacts while allowing control over commanded force, tip stiffness, adapter mass, and input waveform. Compared with hand-held hammering, the actuator reproduces nearly identical strokes under closed-loop control, resulting in much smaller variations in strike timing and peak force between shots [38,39]. Repeatable mechanical impacts are particularly important in elastic-wave testing, where conventional sources often exhibit poor repeatability. Ball and hammer impacts are widely used in impact-echo and acoustic emission testing because the radiated stress waves are directly related to the force–time history of the impact [24,49,50]. However, their impact characteristics are generally less controllable and repeatable than those of the actuator developed in this study. Improved repeatability is practically important because it supports the stacking of repeated shots and enables more reliable comparisons between different experimental conditions.

Across the force tests, increasing the commanded force primarily increases signal amplitude, although a slight shift in the normalized spectra toward lower frequencies is also observed. This trend is consistent with impact mechanics. As the impact becomes stronger, the effective contact time tends to increase, and a longer force pulse naturally contains less high-frequency energy. Impact-echo modeling and Hertzian impact measurements similarly show that the usable upper frequency (or corner frequency) scales inversely with impact duration; therefore, even small increases in contact time can shift spectral energy toward lower frequencies [24,49,51].

Tip stiffness and end mass provide additional means to control the radiated bandwidth. In our experiments, the hard metal tip consistently produces stronger high-frequency energy than the medium tip, consistent with the well-established observation that stiffer contacts generate shorter, sharper force pulses and thus broader bandwidths [24,49,50,51]. Adapter mass also influences the impact response. Increasing the adapter mass enhances the impulse and overall amplitude, but may also lengthen the contact duration and shift spectral energy toward lower frequencies, depending on the contact stiffness [24,51]. In addition to mass, adapter material plays a role in energy transfer. The brass adapter transmits impact energy more efficiently than the 3D-printed plastic adapter. Although slightly heavier, the brass adapter generates a broader frequency spectrum, suggesting that less energy is dissipated within the adapter during impact. This observation agrees with published acoustic-property data showing that yellow brass has a higher longitudinal acoustic impedance and lower intrinsic loss than many plastics, which typically exhibit significantly higher material attenuation [52].

The input waveform and duty cycle also influence impact quality because they determine how the shaft accelerates toward impact and how much time it has to reset before the next cycle. Among the tested waveforms, sine and square commands produce the strongest impacts for the same commanded force. The shaft motion is primarily driven by the impulse delivered during the push portion of the cycle, the area under the force–time curve before contact. With square-wave input, the controller rapidly reaches the commanded force and maintains it for most of the cycle, producing the largest impulse and highest pre-impact velocity. A sine-wave input is smoother but still provides a substantial impulse because the force remains at moderate-to-high levels for a significant fraction of the cycle. In contrast, triangle and sawtooth inputs spend more time ramping through lower force levels, resulting in slower shaft acceleration and lower impact velocity. Consequently, sine and square waveforms generate stronger and more repeatable impacts in our system.

For square-wave operation, each cycle contains an active phase that accelerates the shaft toward impact and an inactive phase that allows the system to decelerate and settle [38,39]. If the inactive period is too short, the system may produce prolonged contact or secondary impacts. Such double-hit behavior is a well-known issue in impact testing because it distorts the force spectrum and can introduce artificial broadband components in frequency-domain analyses [25]. In our experiments, intermediate duty cycles (40% and 72%) produce the cleanest and most repeatable dispersion images, providing sufficient drive time for a stable strike while allowing adequate reset time between cycles.

These source-tuning capabilities are particularly beneficial for guided-wave applications. The $A_{0}$ Lamb mode is strongly dispersive, meaning that different frequency bands correspond to different wavelengths and thus different sensitivity scales when interacting with cracks or other defects [29,37]. Consequently, the ability to control impact bandwidth and repeatability improves the reliability of defect characterization using guided-wave measurements.

### 5.2. The Application of the Actuator on the Concrete Slab with Surface-Breaking Cracks

For the concrete experiments with vertical cracks, a 3 dHz square-wave input with a 72% duty cycle was used, and the tip and commanded force were tuned to reliably excite the $A_{0}$ Lamb mode. The 30 g brass adapter was trimmed to reduce its mass to 20 g, and the white tip was used in all slab experiments. Importantly, these source parameters were kept constant across all crack cases to ensure that the observed anisotropy and attenuation trends reflect the crack response rather than variations in the source. The slab experiments demonstrate that the controllable impact actuator can consistently generate $A_{0}$-dominated wavefields and clear dispersion images in a heterogeneous concrete plate for all three crack depths (Figure 8, Figure 9 and Figure 10). The relatively long wavelengths associated with our concrete thickness naturally average out the scattering effects of small-scale concrete heterogeneities, such as individual aggregates. This provides that the pronounced energy dissipation observed is primarily governed by wave interactions with the vertical cracks. In the time-domain records (Figure 8), the $A_{0}$ wave packet appears more coherent when the sensor array is oriented parallel to the crack. The corresponding frequency–trace maps indicate that the parallel geometry preserves stronger and more continuous energy within the targeted frequency band, whereas the perpendicular orientation produces more localized spectral hot spots. These patterns are consistent with a wavefield that interacts more strongly with the crack faces in the perpendicular configuration. In addition, Figure 10 confirms that phase velocities measured parallel to the crack are systematically higher than those measured in the perpendicular direction. Physically, a surface-breaking crack behaves as a compliant interface; waves crossing the crack experience a displacement discontinuity that introduces additional phase delay and scattering [27,28]. This interpretation is consistent with the linear-slip interface framework, where finite fracture compliance permits such displacement discontinuities under traction [27].

Given that our interested frequency bandwidth is up to 20 kHz, the high-frequency components from 10 to 20 kHz possess wavelengths that are significantly shorter than the concrete slab thickness. In this regime, the $A_{0}$ mode wave transitions from a guided Lamb wave to Rayleigh wave. This transition to behavior of Rayleigh wave is beneficial for this application because Rayleigh wave possesses meaningful energy within one wavelength of the free surface, which makes the anisotropy parameter more sensitive to the surface-breaking cracks. Nonetheless, we still maintain the $A_{0}$ mode designation to reflect the continuous dispersion branch traced from the low-frequency region. Another point is that the HRLRT operates as a global array-processing technique, any non-propagating evanescent modes or body-wave interference captured by the initial sensors in the near-field region tend to be mapped out as uncorrelated background energy, thereby preserving the clarity and resolution of the dispersion images even at lower frequencies.

Crack depth further controls the strength of the directional contrast between parallel and perpendicular measurements. The 6 cm and 12 cm cracks produce relatively similar *η*, whereas the 18 cm crack (approximately 75% of the 24 cm slab thickness) exhibits a substantially larger anisotropic response. This behavior reflects the nonlinear sensitivity of guided waves to crack depth. Increasing the crack depth from 6 to 12 cm increases scattering but does not fundamentally alter the wave–defect interaction. However, when the crack becomes deep and the remaining ligament becomes thin, the interaction intensifies. This observation is consistent with the results of Li and Fromme [28], who showed that for incident $A_{0}$ waves, the mode-conversion amplitude is non-monotonic and reaches a maximum when the defect depth approaches approximately 75% of the plate thickness. At this depth, the remaining ligament is sufficiently thin to maximize wave interaction, which agrees with our observation that the 18 cm crack produces substantially stronger disruption than the shallower 6 cm and 12 cm defects.

The lateral measurements performed along the 18 cm crack (Figure 9g–j) show a systematic spatial trend. The extracted $A_{0}$ phase velocity is lowest when the source–sensor line crosses the central portion of the crack. As the measurement line moves toward the crack tip, the apparent phase velocity increases. The central portion of the crack behaves as a stronger and more extended discontinuity, producing stronger reflections and scattered energy from the crack faces [28]. Closer to the crack tip, the wavefield becomes dominated by diffraction and interference among reflected, transmitted, and mode-converted components, which is typical for finite cracks under oblique Lamb-wave incidence [53,54,55]. These effects modify the dispersion energy distribution, causing the HRLRT ridge and the corresponding phase-velocity picks to vary with lateral position [53,55]. Consequently, the dispersion curve measured approximately 5 cm away from the crack tip exhibits the highest phase velocities among the lateral measurements, with values approaching those of the parallel-array configuration.

The Bootstrap attenuation analysis shown in Figure 11 and summarized in Table 1 indicates that the attenuation estimates are sufficiently stable for interpretation, while acquisition parameters for the impact source and filtering band were held strictly constant across all tests. The shift in the recorded central frequencies in Table 1 varies because of the physical consequence of the crack acting as a frequency-dependent filter, selectively scattering and mode-converting specific wavelengths based on its depth and orientation, thereby altering the frequency content of the surviving transmitted wave packet. By statistically resampling the amplitude decay, the Bootstrap regression inherently downweighs localized outliers that fall within the near-field region, ensuring that the global spatial decay trend is robust against near-field corruption. However, the estimated Q values should be regarded as effective attenuation parameters for the $A_{0}$ wave packet rather than pure material damping values because concrete is heterogeneous, and the measured amplitude decay reflects geometrical spreading, intrinsic absorption, and scattering losses from aggregates, pores, interfaces, and damage [26,56,57]. Consequently, it should not be interpreted in isolation for defect characterization. Instead, its systematic correlation with phase-velocity anisotropy provides the robust, multi-parameter evidence necessary to link these attenuation trends to vertical crack depth. Relative to the intact slab, the cracked configurations exhibit larger β values and lower Q values, indicating faster decay of the coherent $A_{0}$ amplitude once the wavefield interacts with the crack. Furthermore, Q values obtained from the parallel measurements are consistently higher than those from the perpendicular measurements. This observation complements the dispersion analysis: phase velocity primarily reflects changes in effective stiffness, whereas Q characterizes the loss of wave energy and coherence during propagation.

For the deepest crack (18 cm), the Q values are substantially lower than those for the shallower cracks (6 and 12 cm), indicating that a deep defect removes more energy from the propagating $A_{0}$ wave packet and causes the propagation path to behave more like a strongly scattering medium than a clean guided-wave path. The lateral measurements suggest that Q varies along the crack. Near the crack tip, the wavefield becomes more complex because the deep vertical crack generates not only reflection and transmission but also tip diffraction and mode conversion, followed by interference among the scattered components [28,55,58]. Consequently, the $A_{0}$ amplitude decay becomes steeper and the estimated attenuation increases; for example, the Q value near the crack tip (Q ≈ 1.95) remains lower than that measured approximately 5 cm inside the crack region (Q ≈ 3.49). Farther from the crack tip (about 5 cm outside the crack), the receiver path traverses more intact material. Wavenumber-based guided-wave studies show that additional wavenumber components generated by a discontinuity are concentrated near the defect and diminish with distance from it, allowing the wavefield to gradually recover toward the intact-plate response [59,60].

It is important to note that the manufactured cracks in this study represent idealized planar discontinuities with relatively smooth boundaries. Nonetheless, real service-induced cracks usually exhibit tortuosity, variable aperture, roughness, and partial face contact caused by aggregate interlock. These complexities would likely produce additional scattering, localized mode conversion, and spatially variable attenuation. Partial contacts between crack faces may also reduce the effective compliance contrast and therefore decrease the measured anisotropy ratio relative to the idealized cracks examined here. Predicting the exact depth of an unknown crack is a more challenging problem. While this study demonstrates that crack depth directly affects phase-velocity anisotropy and Q-factor, inferring the exact depth in real-world applications will likely require combining data from this active sensing system with numerical modeling.

## 6. Conclusions

In this study, we deployed a controllable mechanical source (an impact actuator) to characterize surface-breaking vertical cracks in a concrete slab. Laboratory validation showed that the impact actuator generated stable impacts with tunable frequency content by adjusting force level, tip stiffness, adapter weight/material, and the driving waveform, while the duty cycle exhibited a comparatively minor variation within the tested range. These settings were optimized to reliably excite the $A_{0}$ Lamb mode on the concrete site. Using dispersion images produced by HRLRT, we extracted the $A_{0}$ phase-velocity curve for cracks at depths of 6, 12, and 18 cm. The results showed a clear anisotropic effect: phase velocities measured parallel to the cracks are higher than those measured perpendicular to them. Crack depth also controls the magnitude of anisotropy. The 18 cm crack (75% of the slab thickness) exhibited the highest anisotropy ratio, while the 6 and 12 cm cracks showed similar values, with the 12 cm crack being slightly higher. Lateral measurements along the 18 cm crack further showed position-dependent dispersion trends, with higher velocities as the array was moved toward the crack tip. Quality factor analysis provided a complementary indicator. Bootstrap-based estimates showed that the presence of a crack leads to a reduction in the quality factor. In general, quality factor values are lower for measurements acquired perpendicular to the crack strike than for measurements parallel to the crack strike. For the 18 cm crack, the lateral measurement taken perpendicular to the crack at its center produced an unstable Q value due to strong scattering effects. The lowest Q was observed at a location 10 cm from the crack tip, and the crack-tip measurement yielded a lower effective Q than the measurement taken 5 cm within the crack region. Nonetheless, measurements taken outside the crack region showed a recovery in effective Q. Overall, phase velocity and effective Q capture different aspects of the crack response, namely, stiffness-related changes and energy loss, respectively. Therefore, using both approaches along with the newly developed source establishes a foundational baseline for crack characterization under controlled environments. However, extrapolating these findings to real-world civil infrastructure requires addressing significant physical complexities not captured in this study. Future work must extend this directional anisotropy framework to evaluate its viability in reinforced concrete environments, where reinforcing steel and coarse aggregates introduce severe multipathing, scattering, and attenuation. Future experimental and numerical studies must investigate the acoustic response of natural, service-induced cracks, which are characterized by irregular geometries, variable apertures, and partial face contact.

## Figures and Tables

**Table 1:** Estimated Q values for different cracks and data acquisition arrays.

Location	Array	Central Freq (Hz)	V (m/s)	*β* Mean (95% Confidence Interval)	*Q* Mean (95% Confidence Interval)
Intact	Parallel	2460	1703.84	0.39 (0.091–1.28)	26.92 (3.55–50.00)
6 cm	Parallel	2480	1807.76	0.68 (0.087–1.87)	11.53 (2.30–49.75)
	Perpendicular	10,340	1688.23	2.50 (1.20–6.75)	10.48 (2.85–16.00)
12 cm	Parallel	7120	1974.07	1.57 (0.37–3.56)	9.74 (3.18–30.73)
	Perpendicular	10,860	1765.81	2.31 (1.31–4.48)	9.09 (4.31–14.72)
18 cm	Parallel	10,660	2031.96	1.89 (1.50–3.48)	9.30 (4.74–11.00)
	Perpendicular	10,840	1452.55	7.14 (3.98–11.64)	3.63 (2.01–5.89)
	10 cm (5 cm from center)	2600	840.36	6.92 (4.71–11.06)	1.45 (0.88–2.06)
	5 cm	9420	1445.03	6.27 (4.02–10.38)	3.49 (1.97–5.09)
	Crack tip	2740	985.76	4.92 (2.032–7.31)	1.95 (1.19–4.30)
	5 cm from the crack	10,840	1721.75	4.76 (1.83–7.20)	4.77 (2.75–10.83)

## Data Availability

The data used in this study are publicly available at [61].

## Supplementary Materials

The following supporting information can be downloaded at: https://www.mdpi.com/article/10.3390/s26144563/s1, Figure S1: Lateral measurement sensor responses and frequency–trace maps for cracked and intact concrete; Figure S2: Bootstrapped fitting results of parameters α and C for varying crack sizes; Figure S3: Bootstrapped fitting amplitudes and parameter distributions *β* and Q for intact concrete and lateral measurements at varying locations; Figure S4: Bootstrapped fitting results of parameters α and C for intact concrete and lateral crack locations; Table S1: Summary of fitting parameters and results for α and C across each recording data.
