# Supplementary material for: Depth-Dependent Characterization of Vertical Cracks in Concrete Using Lamb Wave Active Sensing
Source: Sensors (Basel). 2026 Jul 18;26(14):4563. doi: 10.3390/s26144563 (PMC13417329; doi:10.3390/s26144563)
Supplement: Supplementary file 1 [file sensors-26-04563-s001.zip › sensors-4391542-supplementary.pdf]

Supporting Material for

Depth-Dependent Characterization of Vertical Cracks in Concrete Using Lamb Wave Active Sensing

Nontawat Srisapan[1]*, Theo Asumah[1], Roohollah Askari[1]

[1] Department of Geological and Mining Engineering and Sciences, Michigan Technological University, MI 49931 USA

* Correspondence: Nontawat Srisapan, nsrisapa@mtu.edu

Contents of this file

1) Fig. S1: Lateral measurement sensor responses and frequency–trace maps for cracked and intact concrete
2) Fig. S2: Bootstrapped fitting results of parameters α and C for varying crack sizes
3) Fig. S3: Bootstrapped fitting amplitudes and parameter distributions β and Q for intact concrete and lateral measurements at varying locations
4) Fig. S4: Bootstrapped fitting results of parameters α and C for intact concrete and lateral crack locations
5) Table S1

Introduction

In this document, we provide supplementary figures and a table for the main article. Fig. S1 presents the elastic-wave data from the lateral measurement of the 18 cm crack and the intact concrete along with their corresponding frequency-trace maps. Acoustic waveforms in Fig. S1(a) and (b) are less coherent than those in Fig. S1(c)-(e) due to more interactions with the 18 cm crack, and the waveforms from the intact concrete are the most coherent and uniform. Fig. S2 summarizes the uncertainty in the Bootstrapped fitting parameters for the 6 cm, 12 cm, and 18 cm crack cases,

comparing the parallel and perpendicular sensor configurations. For each case, the histograms show the resulting distributions of the geometric spreading exponent α and the source amplitude C across Bootstrap resamples. The red dashed line marks the mean of each distribution while the black dashed lines indicate the corresponding 95% confidence interval. Overall, the 6 cm and 12 cm cases show more concentrated parameter distributions whereas the 18 cm case exhibits broader and more skewed distributions indicating larger fitting variability for the deepest crack.

Bootstrap fitting results for the intact concrete and the lateral measurements can be found in Fig. S3. In each row, the left plot shows the measured maximum amplitudes (red points) and the best-fit decay curve (black line), with the 95% confidence band from Bootstrapping shaded in blue. The middle and right plots show the corresponding Bootstrap histograms of parameter β and the quality factor Q. Mean values are marked by the red dashed line, and the 95% confidence interval bounds are indicated by black dashed lines. Across the lateral positions (b–e), the fitted β values shift to much larger magnitudes than the intact case (a), and the corresponding Q distributions compress toward low values consistent with stronger attenuation in the vicinity of the crack. Their results for parameter α and C are demonstrated in Fig. S4. Lastly, Table S1 summarizes fitting parameters and results for α and C. Fitting bounds used to run the Bootstrap are also included in the table. Together, these supplementary materials provide additional context for the waveform characteristics and quantify the uncertainty of the attenuation fitting parameters reported in the main text.

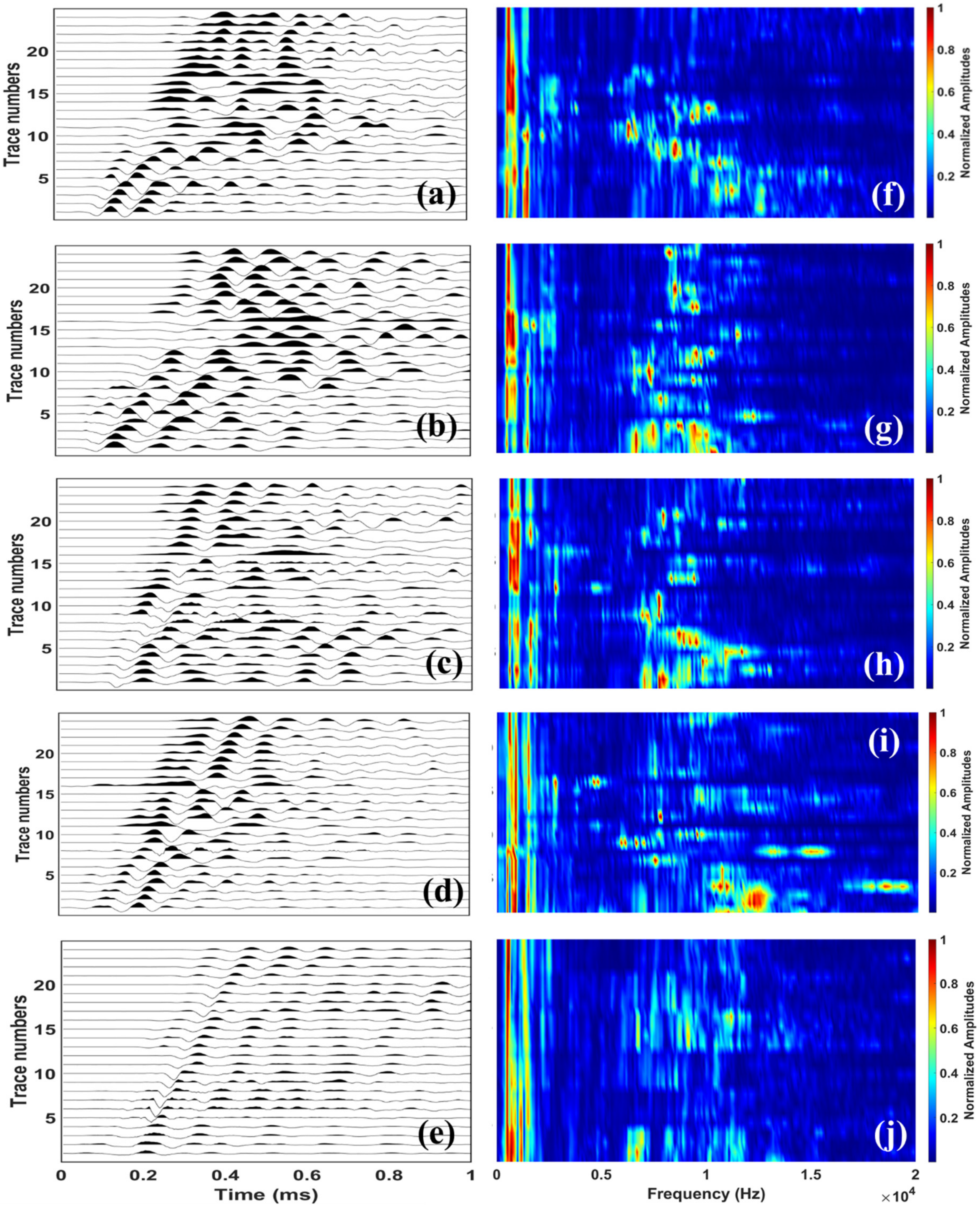

Fig. S1. Lateral measurement sensor responses and frequency–trace maps for cracked and intact concrete: Sensor responses from lateral measurements at the 18 cm crack. Panels (a)-(d) represent locations at 10 cm, 5 cm, crack tip, and 5 cm beyond the crack tip, respectively. Panel (e) shows sensor responses from intact concrete. Panels (f)-(i) show the corresponding frequency–trace maps for (a)-(e), respectively.

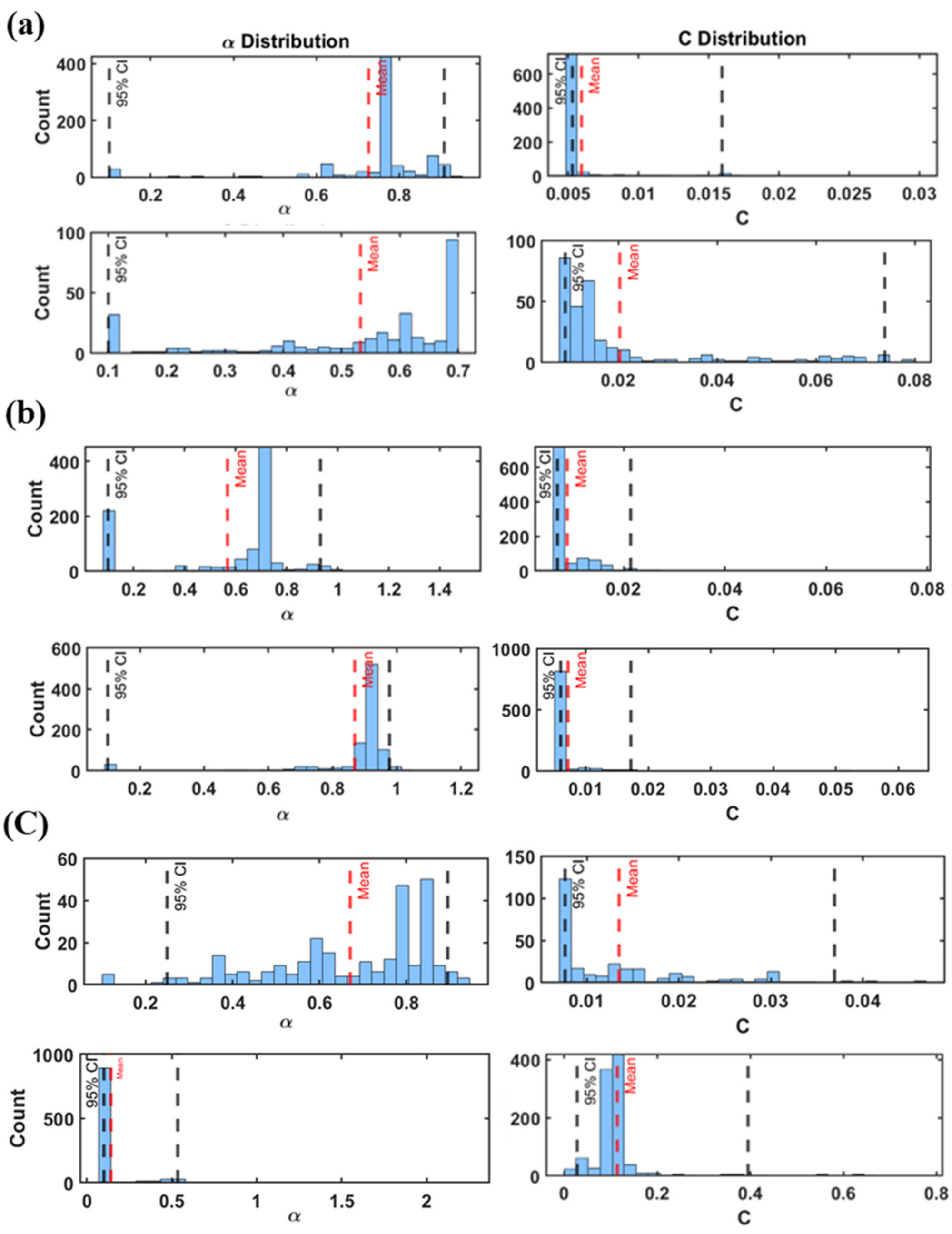

Fig. S2. Bootstrapped fitting results of parameters α and C for varying crack sizes: Bootstrapped fitting results for 6 cm crack (a), 12 cm crack (b), and 18 cm crack (c). Each top row (α and C) represents result from parallel configuration while each bottom row represents perpendicular configuration. Histograms illustrate distributions of α and C, with mean values and 95% confidence intervals indicated by red and black dashed lines, respectively.

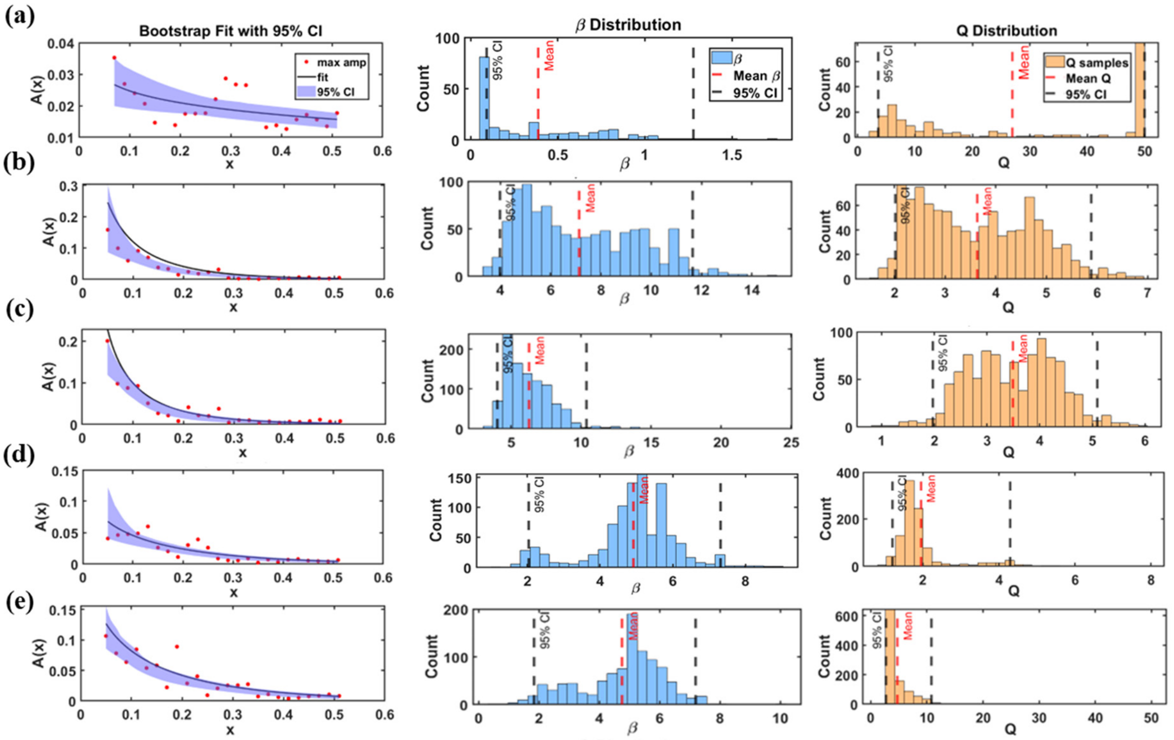

Fig. S3. Bootstrapped fitting amplitudes and parameter distributions β and Q for intact concrete and lateral measurements at varying locations: Row (a), a Bootstrapped fitting results for the intact concrete, and row (b)-(e) Bootstrapped fitting results for lateral measurements of 18 cm crack (10 cm, 5 cm, crack tip, and 5 cm beyond crack tip respectively). The left column represents the fitting amplitudes with 95% confidence interval in blue region, followed by the histogram plots for β and Q distributions. The mean values and 95% confidence intervals indicated by red and black dashed lines, respectively.

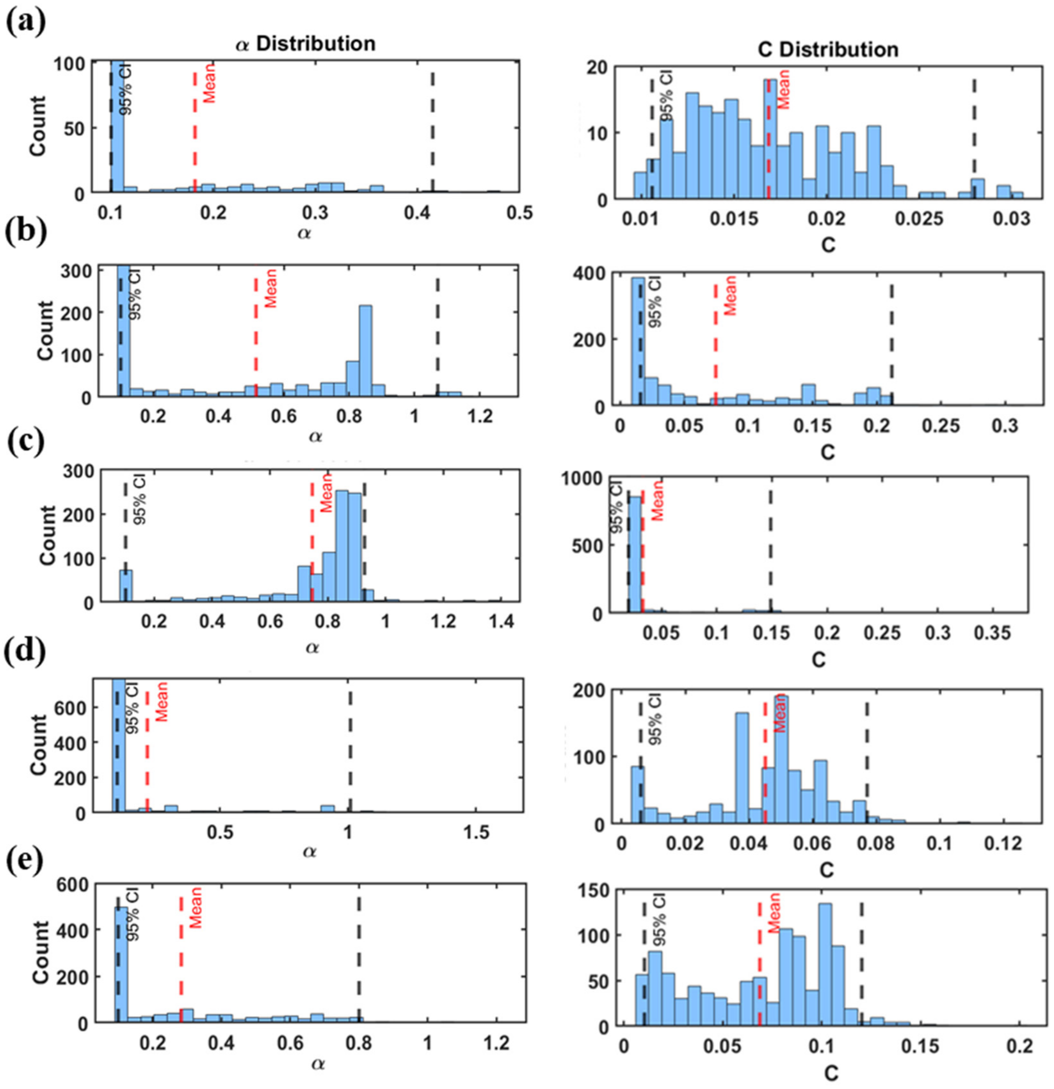

Fig. S4. Bootstrapped fitting results of parameters α and C for intact concrete and lateral crack locations: Bootstrapped fitting results from Fig. S3 for parameters α and C. Row (a) represents intact concrete, while Row (b)–(e) represent lateral measurements of an 18 cm crack at 10 cm, 5 cm, the crack tip, and 5 cm beyond the crack tip, respectively.

Table S1: Summary of fitting parameters and results for α and C across each recording data

Location	Array	Fitting bounds			α Mean (95% confidence Interval)	C Mean (95% confidence Interval)
		α	β	C		
Intact	Parallel	0.10-3.00	0.09-9.07	0.003-0.35	0.18 (0.10-0.42)	0.017 (0.011-0.028)
6 cm	Parallel	0.10-3.00	0.086-8.62	0.005-0.53	0.73 (0.10-0.91)	0.006 (0.005-0.016)
	Perpendicular	0.10-0.70	1.20-38.48	0.007-0.72	0.53 (0.10-0.70)	0.02 (0.009-0.074)
12 cm	Parallel	0.10-3.00	0.23-22.66	0.007-0.68	0.57 (0.10-0.93)	0.009 (0.007-0.021)
	Perpendicular	0.10-3.00	1.29-38.64	0.006-0.59	0.87 (0.10-0.98)	0.007 (0.006-0.017)
18 cm	Parallel	0.10-3.00	1.50-32.96	0.008-0.76	0.67 (0.25-0.89)	0.01 (0.008-0.04)
	Perpendicular	0.10-3.00	0.47-46.89	0.02-1.58	0.52 (0.10-1.07)	0.0747 (0.016-0.21)
	10 cm (5 cm from a center)	0.10-3.00	0.19-19.44	0.013-1.30	0.14 (0.10-0.53)	0.11 (0.028-0.39)
	5 cm	0.10-3.00	0.41-41.00	0.02-2.00	0.75 (0.10-0.93)	0.03 (0.02-0.15)
	Crack tip	0.10-3.00	0.17-17.46	0.006-0.60	0.22 (0.10-1.01)	0.045 (0.006-0.077)

	5 cm from crack	0.10-3.00	0.40-39.56	0.01-1.06	0.28 (0.10-0.80)	0.07 (0.01-0.12)
